# Response Surface Optimization for the Enhancement of the Extraction of Bioactive Compounds from *Citrus limon* Peel

**DOI:** 10.3390/antiox12081605

**Published:** 2023-08-12

**Authors:** Theodoros Chatzimitakos, Vassilis Athanasiadis, Konstantina Kotsou, Eleni Bozinou, Stavros I. Lalas

**Affiliations:** Department of Food Science & Nutrition, University of Thessaly, Terma N. Temponera Str., 43100 Karditsa, Greece; tchatzimitakos@uth.gr (T.C.); kkotsou@agr.uth.gr (K.K.); empozinou@uth.gr (E.B.); slalas@uth.gr (S.I.L.)

**Keywords:** citrus, ultrasound-assisted extraction, pulsed electric field, polyphenols, ascorbic acid, carotenoids, antioxidant activity

## Abstract

*Citrus limon* is among the species of the genus Citrus that dominates the world market. It is highly nutritious for humans as it contains twice the amount of the suggested daily intake of ascorbic acid and is also a good source of phenolic compounds, carotenoids, and other bioactive compounds. This study aimed to identify the optimal extraction procedures and parameters to obtain the maximum quantity of bioactive components from lemon peel by-products. Various extraction techniques, including stirring, ultrasound, and pulsed electric field, were evaluated, along with factors such as extraction time, temperature, and solvent composition. The results revealed that simple stirring for 150 min at 20 °C proved to be the most effective and practical method. The ideal solvent mixture consisted of 75% ethanol and 25% water, highlighting the crucial role of solvent composition in maximizing extraction efficiency. Among the extracted compounds were phenolics, ascorbic acid, and carotenoids. Under optimum extraction conditions, the extract was found to contain high total phenolic content (TPC) (51.2 mg of gallic acid equivalents, GAE/g dry weight), total flavonoid content (TFC) (7.1 mg of rutin equivalents, RtE/g dry weight), amounts of ascorbic acid (3.7 mg/g dry weight), and total carotenoids content (TCC) (64.9 μg of β-carotene equivalents, CtE/g). Notably, the extracts demonstrated potent antioxidant properties (128.9 μmol of ascorbic acid equivalents, AAE/g; and 30.3 μmol of AAE/g as evidenced by FRAP and DPPH assays, respectively), making it a promising ingredient for functional foods and cosmetics. The study’s implications lie in promoting sustainable practices by converting lemon peel into valuable resources and supporting human health and wellness through the consumption of natural antioxidants.

## 1. Introduction

Citrus is a group of plants belonging to the Rutaceae family and the genus Citrus [1]. This diverse group comprises of a variety of fruits, such as orange (*Citrus sinensis*), lemon (*Citrus limon*), mandarin (*Citrus reticulata*), lime (*Citrus aurantifolia*), grapefruit (*Citrus paradisi*), etc. [2]. Among them, *C. sinensis* and *C. limon* are the two most known species [3] with significant economic value [4]. *C. limon*, commonly known as lemon, dominates the global market, but its production is limited to regions with warm and temperate climates due to its sensitivity to low temperatures. Spain leads the production within the Mediterranean region, accounting for 42% of the total, followed by Italy (38%), Greece (5%), and Portugal (1.6%) [5]. Due to their extensive utilization in various ways, lemons have gained popularity. The fruit is primarily used for its juice, while the flesh and peel are used in various culinary applications [6]. This widespread usage can be attributed to the high content of citric acid, which imparts a characteristic sour taste, while they are economically affordable and readily available [7].

Apart from its culinary uses, *C. limon* exhibits valuable nutritional, medical, pharmacological, industrial, and cosmetic properties [8,9]. Notably, lemons are an excellent source of ascorbic acid, containing twice the daily intake needed for an adult (40–45 mg) [10]. This abundance of bioactive compounds, such as phenolic compounds; carotenoids; vitamins A, C, and B; minerals; citric acid; etc., contributes to the many uses and the associated benefits of *C. limon* [11,12]. Lemon juice is well-known for its antimicrobial, antioxidant, antiviral, and anti-inflammatory activities [12,13], while it is also rich in flavonoids, which enhance the immune system and reduce the risk of stroke and cardiovascular diseases [14]. Bioactive compounds with distinct properties are also contained in various parts of *C. limon*. For instance, the dried kernel powder of the fruit contains significant amounts of fiber (60.12 g/100 g), protein (4.89 g/100 g), and ash (3.2 g/100 g) [15]. *C. limon* leaf oil has been found to strengthen the immune, circulatory, and digestive systems, making it valuable for both medicinal and cosmetic purposes, such as removing dead skin cells and cleansing oily skin and hair. Furthermore, the peel of *C. limon* is an effective antioxidant and microbicidal agent, inhibiting the growth of various microbes, including *Escherichia coli*, *Klebsiella* sp., *Staphylococcus epidermidis*, *Staphylococcus aureus*, and *Candida albicans* [16].

Previous studies focused, mainly, on the properties of the essential oil of *C. limon* peel, whereas little attention has been given to the peel itself. Steam distillation has been used as an extraction method to study the antioxidant and antimicrobial capacity of the peel. For instance, steam distillation for 180 min resulted in a 55.1% inhibition of the DPPH free radical and resistance to *Staphylococcus aureus* up to 5% [17]. Similarly, 360 min of steam distillation yielded an antioxidant IC_50_ value of 28.91–37.69 mg/mL enhanced antimicrobial activity against various bacteria and yeasts, including Gram-negative *Pseudomonas fluorescens* [18]. Additionally, aqueous distillation for 300 min resulted in 86.1% inhibition of the DPPH free radical and significant antimicrobial activity against both saprophytic and pathogenic microorganisms [6]. In earlier research, an attempt was made to optimize the extraction of lemon peel in order to ensure a greater amount of citric acid. Five levels of the independent variables, and *X*_1_ (time, 2–45 min), *X*_2_ (sonication power, 50–500 W), and *X*_3_ (ethanol ratio, 0–100%, *v*/*v*) were examined in a response surface methodology. However, the estimation of a final optimal model was not achieved because citric acid was not detected in the peel of all samples [19]. Furthermore, in another study, an effort was made to optimize the extraction conditions of lemon peel. Based on the results of various extraction parameters, results were found that using a drying temperature of 40 °C, methanol at 60% concentration as solvent, an extraction temperature of 60 °C, and an extraction time of 14 min, with which the maximum amount of total polyphenols was obtained. This amount does not exceed 3.55 mg GAE/g d*w* [20].

In the existing literature, there is limited focus on exploring the antioxidant potential of lemon peel. While extensive research has been conducted on the essential oil properties of *Citrus limon* peel, there is a lack of comprehensive investigation into the antioxidant capacity of the peel itself. This gap hinders a deeper understanding of the bioactive compounds present in the peel and their potential applications in various industries. To address the abovementioned research gap, this study aims to unveil the antioxidant potential of lemon peel, thereby providing valuable insights for industrial applications and promoting the sustainable utilization of this by-product. Based on the above, the research hypothesis is that a combination of various extraction techniques, such as pulsed electric field (PEF), ultrasound (US), and stirring (ST), will enhance the antioxidant activity of the extracts obtained from lemon peels. By employing these extraction methods, it is anticipated that a higher concentration of bioactive compounds, including polyphenols, flavonoids, and ascorbic acid, can be obtained from the peel, leading to improved antioxidant capacity. The aim of this study is to optimize the extraction of bioactive compounds, specifically phenolics, carotenoids, and ascorbic acid, from lemon peels. To achieve this aim, a combination of various extraction techniques, including pulsed electric field, ultrasound, and stirring, was employed. The ultimate goal is to contribute to the knowledge about the antioxidant potential of lemon peel, providing valuable information for the development of antioxidant-enriched products in various industries, such as food, pharmaceuticals, and cosmetics. Moreover, the study aims to promote the sustainable utilization of lemon peel as a valuable by-product with significant health and economic benefits.

## 2. Materials and Methods

### 2.1. Chemicals and Reagents

All of the solvents were purchased from Carlo Erba in Val de Reuil, France, and were at least HPLC grade. From Penta (Prague, Czech Republic), we obtained gallic acid; phosphate buffer; anhydrous sodium carbonate; 2,2-diphenyl-1-picrylhydrazyl (DPPH); 2,4,6-tri-2-pyridinyl-1,3,5-triazine (TPTZ); and Folin–Ciocalteu reagent. From Sigma-Aldrich (Taufkirchen, Germany), phenolics (i.e., chlorogenic acid, neochlorogenic acid, cryptochlorogenic acid, rutin, quercetin 3-*O*-galactoside, and kaempferol 3-*O*-rutinoside), aluminum chloride, iron (III) chloride, hydrochloric acid, ascorbic acid, and trichloroacetic acid were purchased as chemical standards. All of the experiments that were performed used deionized water.

### 2.2. Sample and Extract Preparation

*Interdonato* lemons (*Citrus limon*), a high-quality Citrus variety, were provided by local farmers in the province of Corinth (Peloponnese, Greece). After randomly selecting the lemons (14 lemons were randomly selected from a 10 kg pool) one by one, they were placed into a basket. Then, the selected lemons were also divided into two groups of seven lemons (approximately 1 kg), The lemons were washed with tap water and dried with paper towels. Then, the peels were manually removed, cut into smaller pieces and lyophilized in a Biobase BK-FD10P (Jinan, Shandong, China) lyophilizer. The peels were pulverized to a fine powder (with an approximate average particle diameter of about 500 μm) and sieved in an electric sieve of various diameters. The quantity that exceeded 400 μm was selected as an extraction sample and stored in the freezer until further use.

For the extraction process, 20 mL of the extraction solvent and 1 g of the powdered lemon peels were added to a glass beaker. This ratio was adopted after preliminary experiments. The composition of the extraction solvent (% concentration of ethanol in water) is given in Table 1. Extraction was carried out with ST at 500 rpm at various temperatures for varying lengths of time (Table 1). Prior to the extraction step, some samples underwent additional treatments, such as PEF and/or US, as specified in Table 1. Prior to the 20 min treatment with either the PEF and/or US approach, the dried material underwent hydration by being submerged in the solvent for 10 min. The materials were then subjected to the abovementioned ST extraction process. In all cases, after the extraction, the mixture was centrifuged for 10 min at 4500 rpm. The supernatant was retracted and stored at −40 °C until further analysis.

A mode/arbitrary waveform generator (UPG100, ELV Elektronik AG, Leer, Germany), a digital oscilloscope (Rigol DS1052E, Beaverton, OR, USA), a high-voltage power generator (Leybold, LD Didactic GmbH, Huerth, Germany), and two custom stainless-steel chambers (Val-Electronic, Athens, Greece) were used for the PEF processing of the samples [21,22]. The pulse period was set to 1 ms (frequency: 1 kHz), the pulse duration to 10 μs, and the electric field density to 1.0 kV/cm. The Elmasonic P machine (Elma Schmidbauer GmbH, Singen, Germany) was kept at 30 °C and ran at 37 kHz for US treatment.

### 2.3. Design of the Experiment and the Response Surface Methodology (RSM) Optimization

RSM was used to extract the total phenolic content (TPC), total flavonoid content (TFC), ascorbic acid, and total carotenoids with the highest yield achievable. The antioxidant activity was measured using the FRAP, DPPH, and H_2_O_2_ scavenging assays. The objective of the design was to optimize the levels of TPC, TFC, FRAP, DPPH, H_2_O_2_ scavenging, ascorbic acid, and total carotenoids as well as the concentration of neochlorogenic acid. To accomplish this, the extraction process, solvent concentration (*C*, % *v*/*v*), extraction time (*t*, min), and extraction temperature (*T*, °C) were all adjusted. The optimization was based on an experiment using a primary effect screening design and 20 design points. The experimental design required five stages of setup for the process variables. Table 1 contains a list of the coded and real levels. Using the analysis of variance (ANOVA) and summary-of-fit tests, the overall model significance (R^2^, *p*) and the significance of the model (equations) coefficients were evaluated at a minimum level of 95%.

The response variable was also predicted using a second-order polynomial model, as indicated in Equation (1), as a function of the analyzed independent factors:

(1) $$Y_{k}=\beta_{0}+\sum_{i=1}^{2}\beta_{i}X_{i}+\sum_{i=1}^{2}\beta_{\mathrm{ii}}X_{i}^{2}+\sum_{i=1}^{2}\sum_{j=i+1}^{3}\beta_{\mathrm{ij}}X_{i}X_{j}$$

where *Y_k_* is the predicted response variable; *X_i_* and *X_j_* are the independent variables; *β*_0_, *β_i_*, *β_ii_*, and *β_ij_* are the intercept and regression coefficients of the model’s linear, quadratic, and interaction components.

The highest peak area and the impact of a significant independent variable on the response were determined using the RSM.

### 2.4. Determinations

#### 2.4.1. Total Phenolic Content (TPC)

Following a previously established methodology [23], the total phenolic content (TPC) of the extracts was determined using the Folin–Ciocalteu assay. In brief, a 1.5 mL Eppendorf tube was filled with 100 μL of lemon peel extracts and 100 μL of Folin–Ciocalteu reagent. The solution was heated at 40 °C for 20 min before 800 μL of Na_2_CO_3_ solution (5% *w*/*v*) was added. Ultimately, a Shimadzu spectrophotometer (UV-1700, Shimadzu Europa GmbH, Duisburg, Germany) was used to record the absorbance at 740 nm. Gallic acid was used as a standard compound to create a calibration curve. The TPC (*C*_TP_) was measured in milligrams of gallic acid equivalents per liter (GAE). Equation (2) was used to express the extraction yield in terms of total phenolics (*Y*_TP_) as mg GAE per g of dry weight (d*w*):

(2) $$Y_{\mathrm{TP}}\;(\mathrm{mg}\;\mathrm{GAE}/g\;dw)=\frac{C_{\mathrm{TP}}\;\times \;V}{w}$$

where *V* is the volume of the extraction medium (in L), and *w* is the dry weight of the sample (in g).

#### 2.4.2. Total Flavonoid Content (TFC)

The concentration of flavonoids was calculated, based on a previous procedure [24]. 100 μL of the extract was mixed with 860 μL of 35% *v*/*v* aqueous ethanol and 40 μL of a reagent consisting of 0.5 M sodium acetate and 5% *w*/*v* aluminum chloride in an Eppendorf tube. After being vortexed, the resulting mixture was left to react for 30 min at room temperature. The absorbance at 415 nm was then determined. A calibration curve was prepared with rutin solutions (20–100 mg/L) to quantify the concentration of total flavonoids (*C*_TFn_), expressed in mg/L. The TFC was expressed as mg rutin equivalents (RtE) per g of d*w*, using the following Εquation (3):

(3) $$\mathrm{TFC}\;(\mathrm{mg}\;\mathrm{RtE}/g\;dw)=\frac{C_{\mathrm{TFn}}\;\times \;V}{w}$$

where *V* is the volume of the extraction medium (in L), and *w* is the dry weight of the sample (in g).

#### 2.4.3. Ferric Reducing Antioxidant Power (FRAP) Assay

An earlier described technique was used to assess the FRAP [25]. In an Eppendorf tube, 50 μL of the sample extracts were combined with 50 μL of a FeCl_3_ solution (4 mM in 0.05 M HCl), and the combination was then incubated at 37 °C for 30 min. The absorbance at 620 nm was measured following the addition of 900 μL of the TPTZ solution (1 mM in 0.05 M HCl). A calibration curve (*C*_AA_, 50–500 μmol/L in 0.05 M HCl) was created using ascorbic acid as the standard compound. The ferric reducing antioxidant power (*P*_R_) was determined as μmol ascorbic acid equivalents (AAE) per g of d*w*, using the following Equation (4):

(4) $$P_{R}\;(\mu \mathrm{mol}\;\mathrm{AAE}/g\;dw)=\frac{C_{AA}\;\times \;V}{w}$$

where *V* is the volume of the extraction medium (in L), and *w* is the dry weight of the sample (in g).

#### 2.4.4. DPPH Radical Scavenging Activity

An approach that has been used before was used to evaluate the DPPH radicals’ absorption activity [23]. Briefly stated, 975 μL of 100 mM DPPH solution was carefully combined with 25 μL of the sample extract. Following mixing, the solution’s absorbance was measured at 515 nm (*A*_515(*i*)_) and again after 30 min of incubation without light (*A*_515(f)_). The antiradical activity (*A*_AR_) was calculated by employing Equation (5):

(5) $$A_{\mathrm{AR}}\;(\mu \mathrm{mol}\;\mathrm{DPPH}/g\;dw)=\frac{\Delta A}{\varepsilon \;\times \;l\;\times \;C}\;\times Y_{\mathrm{TP}}$$

where Δ*A* = *A*_515(*i*)_ − *A*_515(f)_; ε (DPPH) = 11,126 × 10^−6^ μΜ^−1^ cm^−1^; *C* = *C*_TP_ × 0.025; *Y*_TP_ is the total phenolic yield of the extract (mg/g); and l is the path length (1 cm).

#### 2.4.5. Hydrogen Peroxide (H_2_O_2_) Scavenging Assay

A previously mentioned method was applied for the H*_2_*O*_2_* scavenging assay [26]. In an Eppendorf tube, 400 μL of the extract and 600 μL of a H_2_O_2_ solution (40 mM, made in phosphate buffer, pH 7.4) were added. After 10 min, the absorbance at 230 nm was measured. The capacity to scavenge the H*_2_*O*_2_* was expressed as follows:

(6) $$\%\;\mathrm{Scavenging}\;\mathrm{of}\;H_{2}O_{2}=\frac{A_{o}-(A-A_{c})}{A_{o}}\;\times \;100$$

where *A*_o_, *A*_c_, and *A* are the absorbance of the blank solution, the extract solution in the absence of hydrogen peroxide, and the sample, respectively.

The ascorbic acid calibration curve (*C*_AA_, 50–500 μmol/L in 0.05 M HCl) and the following equation were used to determine the anti-hydrogen peroxide activity (*A*_AHP_) as μmol ascorbic acid equivalents (AAE) per g of d*w*:

(7) $$A_{\mathrm{AHP}}\;(\mu \mathrm{mol}\;\mathrm{AAE}/g\;dw)=\frac{C_{\mathrm{AA}}\;\times \;V}{w}$$

where *V* is the volume of the extraction medium (in L), and *w* is the dry weight of the sample (in g).

#### 2.4.6. Ascorbic Acid (AA) Content

Previous methods were used to evaluate the ascorbic acid (AA) concentration [27,28]. After adding 100 μL of sample extract, 500 μL of 10% (*v*/*v*) Folin–Ciocalteu reagent was added to 900 μL of 10% (*w*/*v*) trichloroacetic acid in an Eppendorf tube. After ten minutes, the absorbance was measured at 760 nm. Ascorbic acid was used to prepare a calibration curve.

#### 2.4.7. Total Carotenoid Content (TCC)

A previously described approach [27] was used to estimate the carotenoid content. 1 g of each sample was extracted by adding 10 mL of ethanol, and it was subsequently stirred at 300 rpm for 30 min at room temperature. Following a 5 min ice bath, during which the mixture was shaken intermittently, the mixture underwent a 5 min 3600× *g* centrifugation. A standard β-carotene calibration curve was utilized to measure the extract’s absorbance at 450 nm and quantify its carotenoid concentration.

#### 2.4.8. HPLC-Based Determination of the Eriocitrin Content and Other Phenolic Compounds

An HPLC system was used to evaluate the sample extracts [27,28]. The study was conducted using Shimadzu CBM-20A liquid chromatography and a Shimadzu SPD-M20A diode array detector (both supplied by Shimadzu Europa GmbH in Duisburg, Germany). The compounds were separated using a Phenomenex Luna C18(2) column from Phenomenex Inc. in Torrance, California, maintained at 40 °C (100 Å, 5 μm, 4.6 mm × 250 mm). A 0.5% aqueous formic acid solution and a 0.5% formic acid in acetonitrile/water (6:4) solution made up the mobile phase, respectively. The gradient program that was used was as follows: 0% B to 40% B, 50% B to 70% B for 10 min, and then remained steady for another 10 min. The mobile phase flowed at a rate of 1 mL/min. After identifying the compounds using the retention time and absorbance spectrum in comparison to pure chemical standards, the quantities were determined using calibration curves (0–50 g/mL). In all cases, the correlation coefficient (R*^2^*) of the prepared plots was above 0.9980. All injections were carried out in triplicates, and the relative standard deviations (RSD) were below 5%. Blank samples were also analyzed to confirm that no interfering peaks overlapped with the analyte peaks of interest.

### 2.5. Statistical Analysis

The experimental design, statistical analysis relevant to the response surface methodology, and distribution analysis were all performed using JMP^®^ Pro 16 software (SAS, Cary, NC, USA). The quantitative analysis was performed in triplicate, and the extraction processes were performed at least twice, once for each lemon batch. The results are shown as medians and standard deviations.

## 3. Results and Discussion

Prior to conducting this study, the physical and chemical parameters of the lemons were carefully considered to ensure that the lemons used herein were at the appropriate maturity stage. The fruit limbs and branches exhibited a vibrant green color, while the epicarp displayed a characteristic yellow hue. The weights of the fruits ranged from 50 to 80 g. The titratable acidity (TA) (reported as % citric acid) was 6.59 ± 0.08, the pH value was 2.39 ± 0.02, and the total soluble solid (TSS) (°Brix) value was 9.83 ± 0.06. The TSS/TA ratio was used to calculate the Sweetness Index (SI), which was calculated to be 1.5, indicating a low sweetness level. The TA/TSS ratio was used to calculate the Astringency Index (AI), which was found to be 0.7. A high level of acidity in lemons typically signifies their ripeness, and this was demonstrated by the acidic pH and the low sweetness level measured in the fruit before harvesting. The above values are consistent with earlier report [29].

### 3.1. Extraction Optimization

To ensure that the extracts obtained from the lemon peels contained the maximum amount of bioactive compounds, the RSM approach was employed. This method made the systematic exploration of multiple factors affecting the extraction process feasible, including the extraction methods (ST, US, PEF, and their combinations), as well as the extraction parameters, such as the extraction time (ranging between 15 and 150 min), extraction temperature (ranging from 20 to 80 °C), and solvent composition (water; ethanol; and their mixtures of 25, 50, and 75% *v*/*v*). Based on previous research and preliminary experiments, the ideal solid-to-solvent ratio of 1:20 (1 g of lemon peels to 20 mL of solvent) was determined [26].

**Figure 1:**
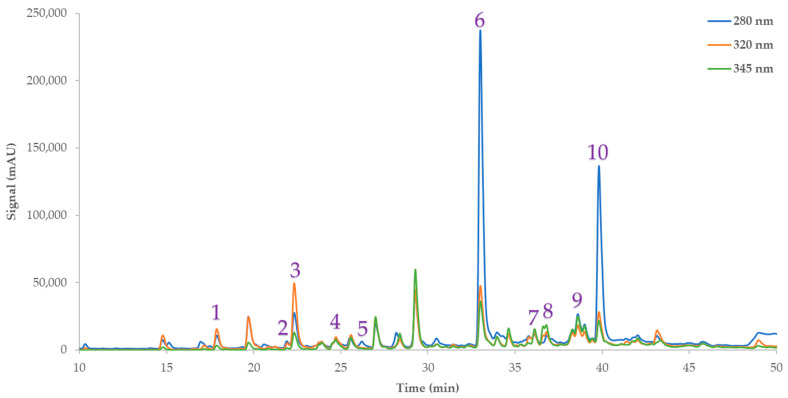
Representative HPLC chromatogram at 280, 320, and 345 nm of Citrus peels extract, demonstrating phenolic compounds that were identified. 1: Neochlorogenic acid; 2: catechin; 3: chlorogenic acid; 4: caffeic acid; 5: syringic acid; 6: eriocitrin; 7: luteolin 7-glycoside; 8: kaempferol 3-*O*-β-rutinoside; 9: kaempferol 3-glycoside; 10: hesperidin.

Using the RSM approach, the impact of each factor was assessed and tuned, so as to achieve maximum extraction efficiency. The measured responses for each extract are presented in Table 2. As can be seen from the results, design point 9 was found to yield the best results compared to the other 19 samples. This sample recorded optimal or near-optimal values in all measured responses. Among the identified compounds, eriocitrin emerged as the major phenolic compound in the extracts, with concentrations ranging from 5.08 to 7.10 mg/g. The extracts were analyzed using HPLC-DAD. Results are presented in Table 3, and a representative chromatogram is given in Figure 1. Various phenolic compounds, such as hesperidin (2.26–6.44 mg/g), caffeic acid (0.04–0.09 mg/g), luteolin 7-glycoside (0.13–0.17 mg/g), chlorogenic acid (0.4–0.76 mg/g), neochlorogenic acid (0.02–0.18 mg/g), catechin (0.04–0.36 mg/g), syringic acid (0.01–0.07 mg/g), kaempferol 3-*O*-β-rutinoside (0.21–0.33 mg/g), and kaempferol 3-glycoside (0.38–0.44 mg/g), were detected and quantified in the samples.

Hesperidin is one of the main flavonoids present in lemons and sweet oranges [30]. Comparing our results to a previous study on *C. sinensis* peels, in which different methanolic extracts contained a maximum of 0.36 mg/g of hesperidin, our extracts displayed a notably higher concentration, since even the extract with the lowest concentration contained 2.26 mg/g (527% higher content) [31]. Similarly, increased quantities of caffeic acid, ranging from 0.04 to 0.09 mg/g, were found in our extracts, compared to its presence in various parts of *C. limon*, particularly in the pulp (about 0.02–0.13 mg/g) and the whole fruit (about 0.13–0.30 mg/g) [32]. Chlorogenic acid, the second most abundant phenolic acid in *C. limon*, was also significantly increased by up to 448% in our extracts, compared to a previous study [32]. Luteolin 7-*O*-glucoside is known to exist in very low concentrations in *C. limon* (0.02%) [33]. However, our study demonstrated that by applying the extraction parameters, the concentration of luteolin 7-*O*-glucoside can increase by up to 17%. A similar increase in the content of neochlorogenic acid was also recorded in our case, since the extracts were found to contain quantities of up to 0.18 mg/g, higher than the value of almost 0.13 mg/g reported in a previous study [34]. Polyphenols, such as catechin and syringic acid, were rarely studied in *C. limon*, but our results indicated their presence in the peel extracts, with quantities reaching up to 0.39 mg/g for catechin and quantities ranging from 0.01 to 0.08 mg/g for syringic acid. Additionally, kaempferol 3-*O*-β-rutinoside, a flavonoid frequently detected in different parts of *C. limon*, such as the zest and juice [35,36,37], was found in variable amounts (0.21–0.33 mg/g) in the peel extracts, making it one of the most abundant flavonoids after eriocitrin, hesperidin, and chlorogenic acid. Astragalin (kaempferol-3-glycoside), another flavonoid with diverse biological properties, was also detected in *C. limon* peel extracts, with a substantial amount of 0.44 mg/g, further supporting the high content of flavonoids in this part of the fruit [38].

The optimization of the extraction parameters in our study was of high significance as it achieved maximum extraction efficiency while minimizing the consumption of energy, time, and solvents. Optimizing these parameters is essential for reducing the environmental impact of the entire extraction process [26]. The choice of solvent composition significantly affects compound extraction [22]. For instance, bioactive compounds with medium polarity, such as the polyphenols abundantly identified in our samples, are not effectively extracted with water, due to their lower polarity [27]. Therefore, ethanol, has been widely used to improve extraction efficiency [21,23,39]. In our case, a concentration of 75% *v*/*v* ethanol proved to be most effective in increasing extraction yield, resulting in the optimal extraction of polyphenols and other nutritional compounds. Although green extraction techniques are generally considered more efficient, our experimental results revealed that simple stirring was sufficient to achieve the maximum extraction of all bioactive compounds. Furthermore, high temperatures seemed to act as an inhibiting factor for achieving the optimal concentration of any bioactive compound.

**Figure 2:**
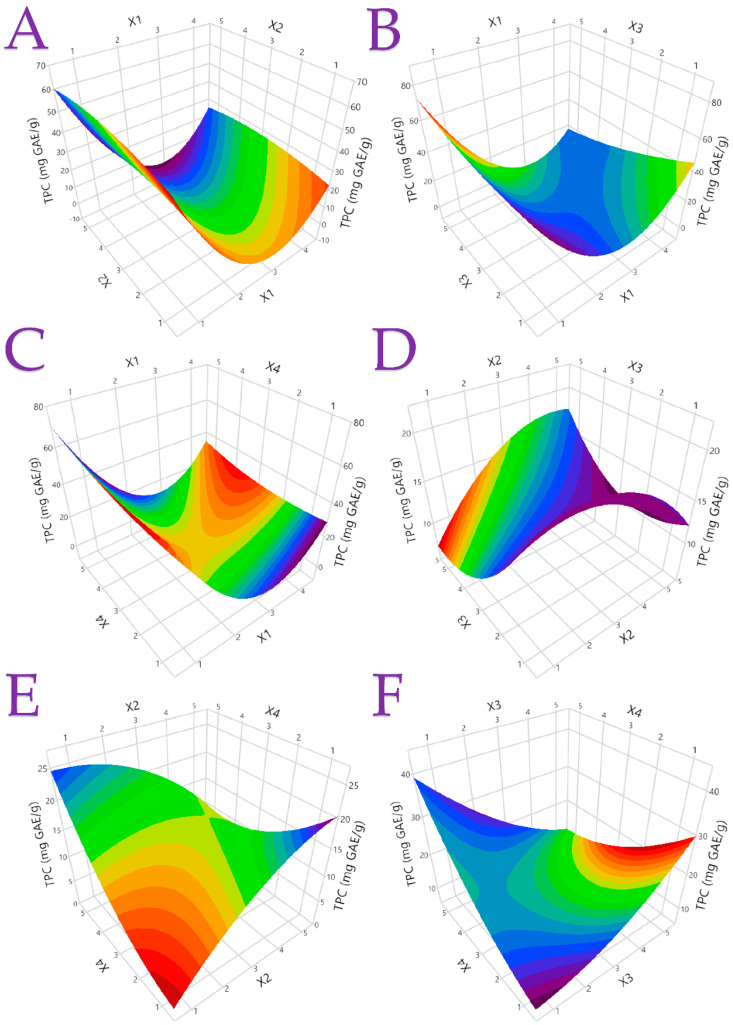
The optimal extraction of Citrus peel by-product extracts using different extraction methods and hydroethanolic solutions is shown in 3D graphs that show the impact of the process variables considered in the response (total phenolic content (TPC), mg GAE/g). Plot (**A**), covariation of *X*_1_ and *X*_2_; plot (**B**), covariation of *X*_1_ and *X*_3_; plot (**C**), covariation of *X*_1_ and *X*_4_; plot (**D**), covariation of *X*_2_ and *X*_3_; plot (**E**), covariation of *X*_2_ and *X*_4_; and plot (**F**), covariation of *X*_3_ and *X*_4_.

**Figure 3:**
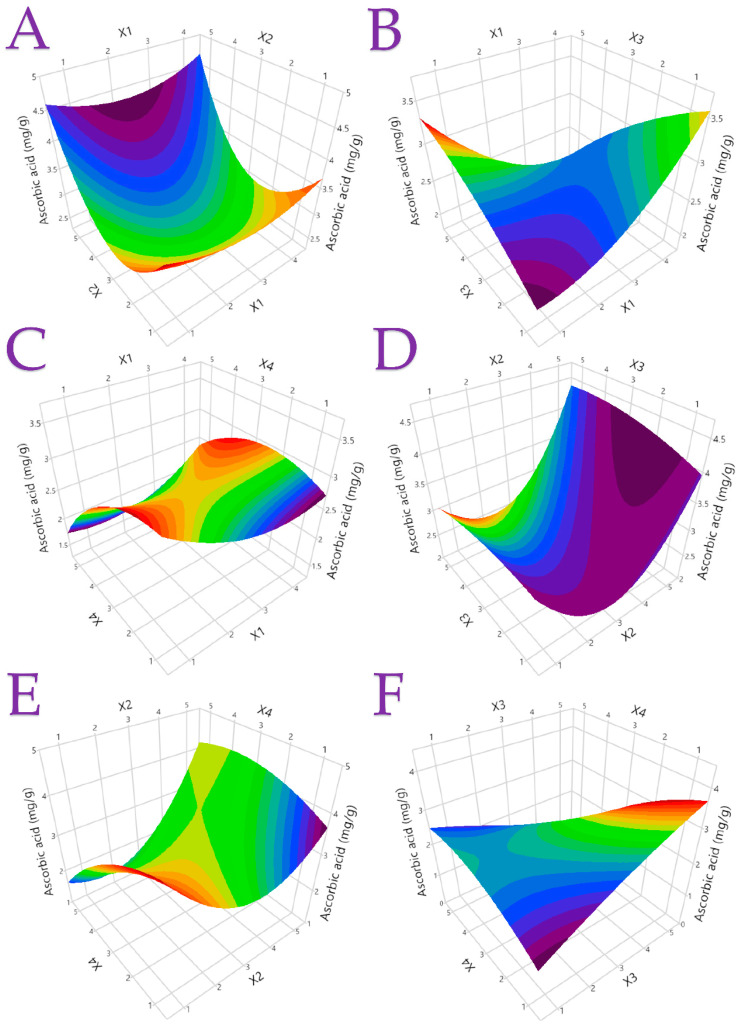
The optimal extraction of Citrus peel by-product extracts using different extraction methods and hydroethanolic solutions is shown in 3D graphs that show the impact of the process variables considered in the response (ascorbic acid, mg/g). Plot (**A**), covariation of *X*_1_ and *X*_2_; plot (**B**), covariation of *X*_1_ and *X*_3_; plot (**C**), covariation of *X*_1_ and *X*_4_; plot (**D**), covariation of *X*_2_ and *X*_3_; plot (**E**), covariation of *X*_2_ and *X*_4_; and plot (**F**), covariation of *X*_3_ and *X*_4_.

The statistical parameters, second-order polynomial equations (models), and coefficients (>0.95), generated for each model are presented in Table 4, indicating well-fitting models. Figures S1–S8 display plots of the actual response compared to the expected response for each investigated parameter, along with the desirability functions. Figure 2 and Figure 3 provide three-dimensional response plots for TPC and ascorbic acid, while Figures S9–S14 present three-dimensional response plots for additional responses. These comprehensive results serve as valuable insights for the optimization of the extraction processes and the effective utilization of the bioactive compounds from lemon peels in various applications.

### 3.2. Analysis of the Extracts

#### 3.2.1. Total Phenolic Content (TPC) and Flavonoid Content (TFC) of the Extracts

The TPC and TFC in the extracts was significantly influenced by the chosen extraction techniques and parameters, as presented in Table 2. The TPC ranged from 5.1 mg GAE/g to 51.2 mg GAE/g, while the TFC varied from 2.5 mg RtE/g d*w* to 7.1 mg RtE/g d*w*. Optimal extraction yields of phenolics were achieved when using a combination of ethanol with an extended extraction time (Table 5). Interestingly, elevated temperature and other extraction techniques, prior stirring, did not further enhance the TPC and TFC. In a previous study aiming to optimize the extraction of bioactive compounds, a hot water extraction method under pressure was employed, with the temperature ranging from 40 °C to 200 °C. The optimum TPC of 10.1 mg GAE/g was achieved at 40 °C, while the TFC increased from 1.4 mg CE/g at 40 °C to 6.2 mg CE/g at 200 °C [40]. While comparing the results and techniques proposed in that study to our findings, it is evident that employing the proposed energy-efficient techniques, such as ST and PEF in combination with ethanolic solvents (100% ethanol and 50% ethanol-water), can ensure higher amounts of these bioactive compounds. In our study, the TPC were found to be increased by 406% and TFC increased by 14% compared to the maximum amounts reported in that study. According to Table 6, the values predicted by Partial Least Squares (PLS) for the optimal extraction would be 47.8 mg GAE/g for TPC and 5.4 mg RtE/g for TFC. However, based on the maximum values observed in Table 5, the actual predicted responses are 51.2 ± 14.7 mg GAE/g for TPC and 7.5 ± 1.4 mg RtE/g for TFC. These findings demonstrate that the careful selection of extraction techniques and solvent composition can significantly enhance the extraction efficiency and yield of bioactive compounds, offering a more energy-efficient and effective approach.

#### 3.2.2. Eriocitrin Content and Other Phenolics of the Extracts

Eriocitrin, a phenolic compound abundant in the citrus family, is particularly found in high amounts in *C. limon* juice and peels [41]. This bioactive compound has been extensively studied for its diverse biological and pharmacological actions. It shows promise in regulating lipid concentrations, displaying antidiabetic properties, providing cytoprotective effects, and reducing inflammatory factors, such as NF-B, TNF-, IL-1, and IL-6, which are associated with various diseases. Additionally, eriocitrin influences the MAPK and STAT3 pathways in specific tumors and cancers, like hepatocellular carcinoma, promoting the expression of genes related to apoptosis [42]. Among the twenty samples, the extraction conditions that led to the maximum concentration of eriocitrin in *C. limon* peel extract was carried out using a solvent containing 25% ethanol, and the extract was prepared through ST at a high temperature of 80 °C for a period of 150 min. This optimized approach yielded the highest eriocitrin extraction. As for the optimal amount of individual polyphenols, such as hesperidin, chlorogenic acid, and others, that can be obtained by using the suggested PLS parameters, the results are given in Table 7 (vide infra). A noteworthy outcome is that the concentrations of eriocitrin and hesperidin are quite close. In a previous study, eriocitrin was examined in different parts of the *C. limon* plant, with concentrations of 0.37 ± 0.11 mg/g, 0.17 ± 0.02 mg/g, and 0.06 ± 0.01 mg/g found in young leaves, young stems, and flowers, respectively [11]. In our case, significantly higher quantities of eriocitrin (ranging from 5.1 to 7.0 mg/g) were successfully extracted from the lemon peel samples. Specifically, the maximum amount obtained was 7.2 ± 0.2 mg/g d*w* (Table 5). The high increase in eriocitrin content achieved through the proposed optimized extraction process suggests that lemon peels can serve as a valuable source of this bioactive compound. Given the various health-promoting properties of eriocitrin, its enhanced extraction from *C. limon* peels holds great potential for applications in the nutraceutical and pharmaceutical industries. The results of this study contribute valuable information for the further exploration of eriocitrin’s diverse health benefits and the development of innovative products with enhanced nutritional and therapeutic properties.

#### 3.2.3. Antioxidant Properties of the Extracts

The antioxidant properties of the extracts obtained in this study were evaluated using three different methods (i.e., FRAP, DPPH, and H_2_O_2_ reduction). *C. limon* antioxidant activity has been previously studied [43]. In a previous study, the antioxidant properties of *C. limon* peel extract were assessed. By comparing the results of this study to ours, the significant role of ethanol in isolating the highest amount of antioxidants is evident, as well as the influence of a lower room temperature. However, our study demonstrated that simple stirring was sufficient to achieve optimal results, in contrast to the previous study which required the use of ultrasound at 300 W, resulting in lower amounts of antioxidants [44]. More specifically, antioxidant values of 18.6 μmol TE/g (FRAP) and 16.5 μmol TE/g (DPPH) [44] were previously reported, while in our case, values of 128.9 μmol AAE/g (FRAP) and 30.3 μmol AAE/g (DPPH) were recorded. The fact that US was not found to promote the extraction of bioactive compounds may be due to the temperature increase that takes place during US within the cytoplasm, which can lead to the degradation or decomposition of some phytochemical compounds, especially those that are sensitive to high temperatures or radical-induced reactions. Our findings are in line with other research where a citrus-based juice blend reduced H_2_O_2_-induced cellular damage and the intracellular production of reactive oxygen species in human dermal fibroblasts [45]. Additionally, the addition of lemon to tea significantly reduced H_2_O_2_ production [46]. These results align with those of our study, as we observed the strong inhibition of H_2_O_2_ by using ethanol as a solvent, conducting the extraction under room temperature for an extended duration, and employing ultrasound prior to stirring. According to Table 5, the maximum predicted values for each method of measuring antioxidant capacity were 128.9 ± 33.5 µmol AAE/g for FRAP with a desirability of 0.89, compared to the maximum value of 1. For DPPH, the value was 30.3 ± 3.5 μmol AAE/g, with a desirability of 0.995, while for H_2_O_2_, the value was 44.7 ± 8.7 μmol AAE/g, with a desirability of 0.976.

#### 3.2.4. Ascorbic Acid Content of the Extracts

Ascorbic acid, a natural antioxidant [47], plays a vital role in regenerating additional antioxidants in the body, including α-tocopherol (vitamin E) [48]. Aside from its antioxidant functions, ascorbic acid also enhances the absorption of non-heme iron [49]. A noteworthy result from our study is the high recovery rate of ascorbic acid from lemon peel. While previous research reported lemon juice contains approximately 0.28–0.52 mg/g of ascorbic acid [50], our results demonstrate a substantial improvement. By subjecting the sample to US treatment followed by ST at room temperature, using 100% ethanol as the solvent, we extracted high amounts of ascorbic acid (3.71 mg/g). In comparison, a previous study examining aqueous extracts of lemon peels reported a significantly lower recovery of ascorbic acid, as low as 0.59 mg/g [14,51]. This striking difference of 533% highlights the pivotal role of the solvent in optimizing the extraction process and effectively isolating valuable nutrients. Our study achieved a maximum amount of 3.88 mg/g d*w* (Table 5) using PEF, attaining a desirability value exceeding 0.9. The successful extraction and substantial recovery of ascorbic acid from lemon peel carry significant implications for various industries. Ascorbic acid, a potent antioxidant, has widespread applications in food, pharmaceuticals, and cosmetics. The efficient extraction process demonstrated in our study opens avenues for developing antioxidant-rich products with potential health benefits.

#### 3.2.5. Total Carotenoid Content (TCC) of the Extracts

For over a century, total carotenoids have been the subject of extensive research across various disciplines, including chemistry, biochemistry, food science and technology, medicine, pharmaceuticals, and nutrition. This interest was driven by the multitude of health benefits carotenoids offer, such as boosting the immune system; protecting against serious diseases, like cancer and cardiovascular diseases; and their renowned anti-aging properties [52]. Our study focused on investigating the TCC in *C. limon* peel extracts, revealing their substantial presence in the as-prepared extracts. Notably, the use of PEF prior to ST proved to be a crucial factor in significantly increasing the maximum TCC in the extracts. Additionally, the extraction time, temperature, and solvent composition emerged as equally important variables. Optimal extraction could be carried out at a moderate to high temperature of 50 °C and a solvent combination of 50% ethanol and deionized water. According to Table 2, the maximum TCC obtained was 64.9 μg CtE/g, while the lowest amount found in the samples was 3.3 μg CtE/g. Notably, by adjusting the extraction conditions, specifically modifying the temperature to 65 °C while keeping the other parameters similar, the maximum predicted value (81.5 ± 12 μg CtE/g) was achieved, nearing a desirability value of 1 (Table 5). These results highlight the importance of optimizing the extraction technique and temperature to achieve a significant yield of total carotenoids from lemon peel. Meanwhile, according to Table 5, by using similar conditions and modifying only the temperature to 65 °C, the maximum predicted value (81.5 ± 12 μg CtE/g) can be achieved with a desirability value close to 1. To further underscore the significance of our findings, we compared them to a previous study on various citrus peels, including *Citrus grandis*, which recorded TCC of 21 and 36 μg/g [53]. Our study demonstrated an enhancement of approximately 80% to 209% in the maximum TCC, further emphasizing the importance of carefully selecting the extraction technique and temperature.

### 3.3. Factor Analysis (FA) and Multivariate Correlation Analysis (MCA)

**Figure 4:**
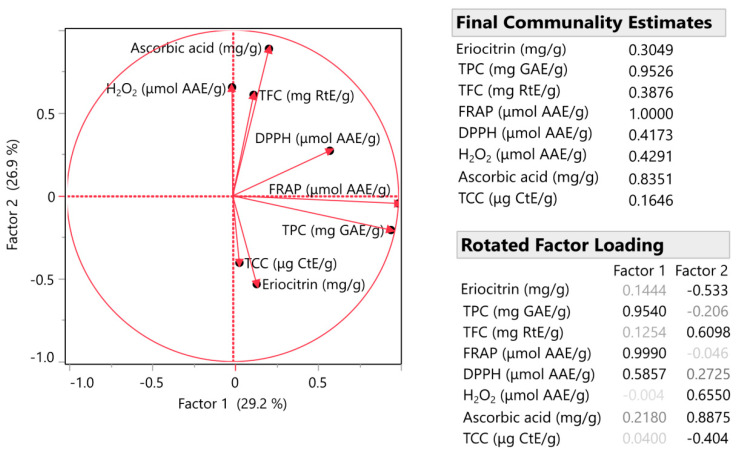
Factor analysis for the measured variables. Inset tables include communality estimates for each variable (eriocitrin content, total phenolic content (TPC), total flavonoid content (TFC), ferric reducing antioxidant power (FRAP) assay, DPPH radical scavenging activity, hydrogen peroxide (H_2_O_2_) scavenging assay, ascorbic acid content and total carotenoid content (TCC)).

**Figure 5:**
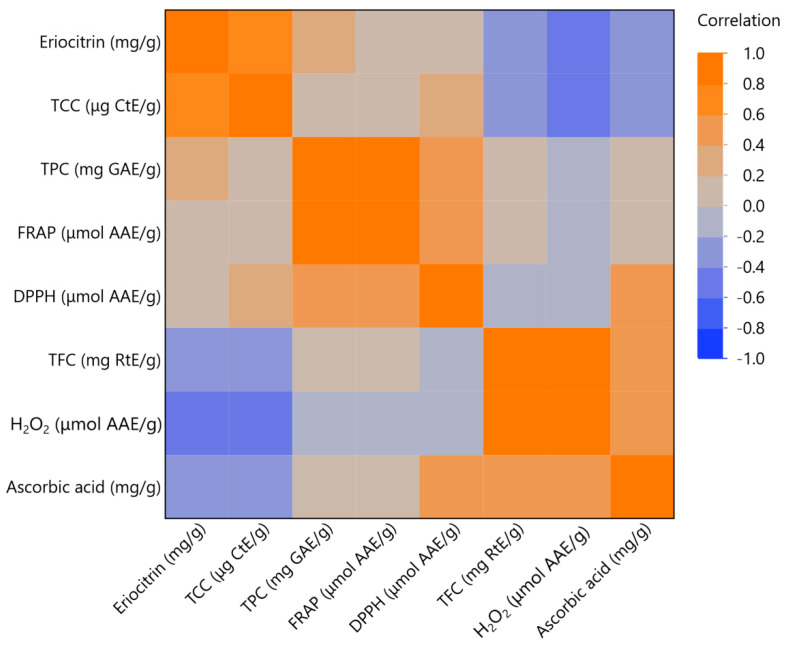
Multivariate correlation analysis of measured variable (eriocitrin content, total phenolic content (TPC), total flavonoid content (TFC), ferric reducing antioxidant power (FRAP) assay, DPPH radical scavenging activity, hydrogen peroxide (H_2_O_2_) scavenging assay, ascorbic acid content, and total carotenoid content (TCC)).

As shown in Figure 4 and Figure 5, important correlations among similar sample variables were highlighted. As the correlation value approaches 1, it signifies a stronger association between the variables. Notably, we observed significant correlations between various bioactive compounds. For example, the eriocitrin content was correlated with the TPC with a correlation coefficient of 0.8, and a similar correlation value was observed between the TPC and DPPH antioxidant capacity. Moreover, the antioxidant properties of the samples, as measured using DPPH and FRAP, also displayed a high correlation of >0.8. A strong correlation was shown between TFC and peroxide production resistance. Finally, ascorbic acid appeared to have a great correlation with the antioxidant capacity of the samples, as expected, as well as with the flavonoids contained in the sample. Such high correlations are rarely achieved. In fact, in many cases, it is observed that the correlation between the variables is much lower, almost half, or even negative [26].

### 3.4. Partial Least Squares (PLS) Analysis

**Figure 6:**
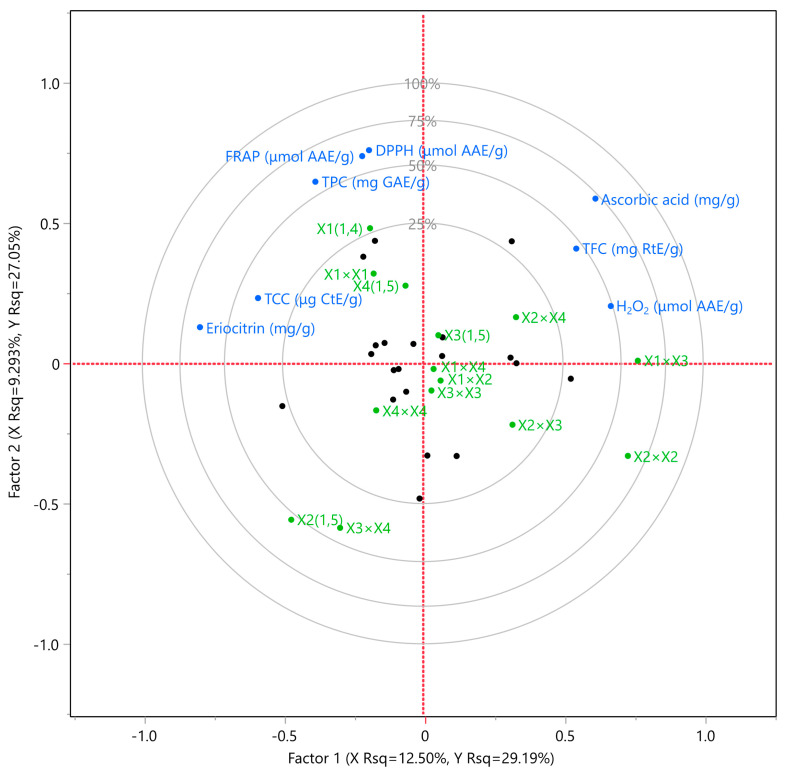
PLS (partial least squares) analysis was used to create graph (correlation loading plot) showing Citrus peel extraction for each variable (eriocitrin content, total phenolic content (TPC), total flavonoid content (TFC), ferric reducing antioxidant power (FRAP) assay, DPPH radical scavenging activity, hydrogen peroxide (H_2_O_2_) scavenging assay, ascorbic acid content, and total carotenoid content (TCC)) using different extraction methods and hydroethanolic solutions.

**Figure 7:**
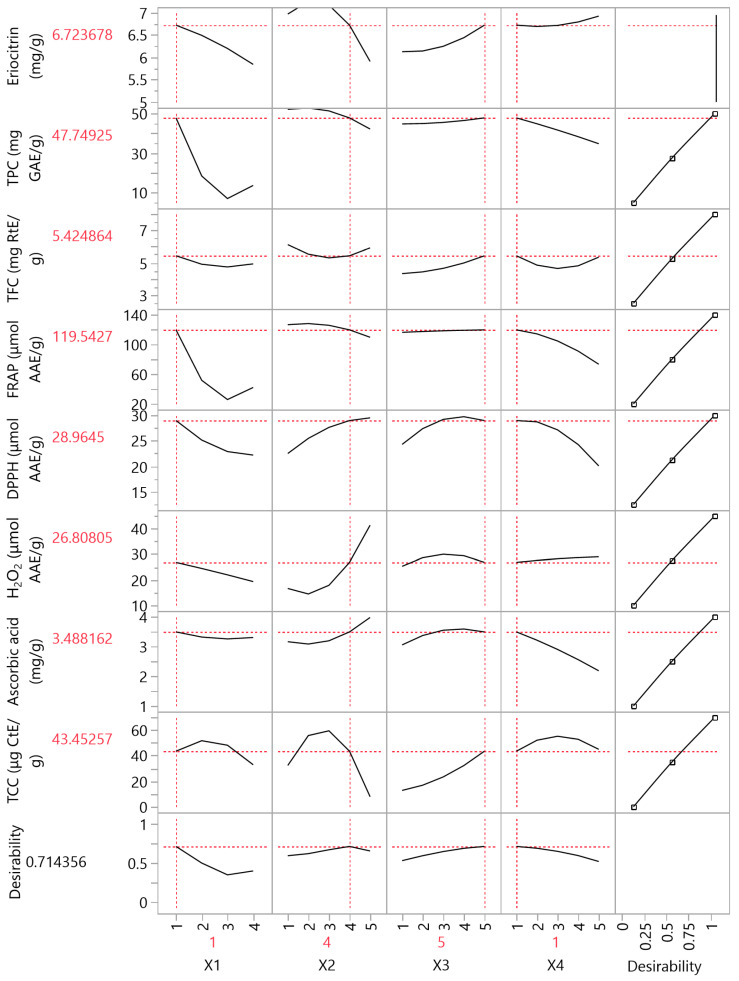
Partial least squares (PLS) prediction profiler of each variable (eriocitrin content, total phenolic content (TPC), total flavonoid content (TFC), ferric reducing antioxidant power (FRAP) assay, DPPH radical scavenging activity, hydrogen peroxide (H_2_O_2_) scavenging assay, ascorbic acid content and total carotenoid content (TCC)) and desirability function with extrapolation control for the optimization of Citrus peel extracts using different extraction methods and hydroethanolic solutions.

A PLS analysis (Figure 6 and Figure 7) was conducted to determine the most important factors among the examined extraction variables (*X*_1_, *X*_2_, *X*_3_, and *X*_4_). According to Figure 6, the extraction method (*X*_1_) and the temperature (*X*_4_) were found to be the most important extraction parameters for polyphenols, carotenoids, and antioxidant activity enhancement. ST was found to be more suitable, offering optimal results in the fastest and most economical way. Moreover, it was observed that higher temperatures did not favor the extraction of all bioactive compounds, and 20 °C was sufficient to achieve maximum results. Regarding the solvent, a high ethanol concentration (at least 75%) was found to ensure optimal results for most cases. Furthermore, the extraction duration also played a critical role in optimizing the extraction conditions, with 150 min being the most prevalent duration. Figure 7 presents the values of all bioactive compounds that can be extracted if the proposed extraction conditions are followed according to PLS. The desirability of results does not appear particularly high, with a recorded value of approximately 0.7144. This difference is likely due to the temperature parameter, as some compounds required a higher extraction temperature (i.e., 80 °C) to reach their maximum value. It is important to note that PLS provides valuable insights into the optimization of extraction conditions and aids in understanding the interplay of various factors affecting the bioactive compound yields.

## 4. Conclusions

The present study identified the optimal extraction procedures and parameters to maximize the yield of bioactive components from lemon peel. Among the various techniques evaluated, ST proved to be the most effective and practical extraction method. ST conducted for 150 min at a temperature of 20 °C, offered a straightforward and cost-efficient approach. Although the extraction process with ST for 150 min can be considered time-consuming compared to some other methods that may offer shorter extraction times, the extended extraction time was indeed a trade-off to achieve higher yields of bioactive compounds using this particular method. Since time efficiency is a significant consideration for practical applications, in real-world scenarios, researchers and industry professionals may weigh the benefits of higher extraction yields against the time consumption to determine the most suitable extraction method for their specific needs. The choice of extraction solvent also played a crucial role in the extraction process. The optimal mixture of 75% ethanol and 25% water achieved the highest extraction efficiency, highlighting the importance of solvent composition. The bioactive compounds extracted from lemon peels, particularly the antioxidant components, hold significant importance in promoting human health. As consumer demand for antioxidant-rich foods and cosmetics grows, our findings present valuable opportunities for further research in this area. By recognizing lemon peels as a valuable source of bioactive compounds, rather than just a by-product in the food industry, there is the potential to transform this by-product into a valuable resource. The efficient extraction of bioactive components from lemon peels using a simple and cost-effective method is a significant step towards sustainable utilization and value addition. The obtained extracts can be utilized in the development of functional foods, nutraceuticals, and cosmetics with enhanced antioxidant properties. This study not only highlights the potential of lemon a by-product, but also calls for further exploration of the practical applications and commercial viability of these bioactive extracts. By harnessing the potential of lemon peels and employing appropriate extraction techniques, the industry can contribute to sustainable practices and capitalize on this valuable resource. In conclusion, the utilization of lemon peels as a source of bioactive compounds presents promising opportunities for innovative and sustainable product development. The knowledge gained from this study can be used by the functional food and cosmetic industries, where the emphasis on natural antioxidants and health benefits continues to rise. Embracing this approach can lead to a greener and more resource-efficient future, enhancing the overall value chain of the food and cosmetic sectors while reducing waste and promoting circular economy practices.

## Figures and Tables

**Table 1:** The actual and coded levels of the independent variables that were used to optimize the process.

Independent Variables	Code Units	Coded Variable Level				
		1	2	3	4	5
Technique	*X*_1_	ST	PEF + ST	US + ST	PEF + US + ST	–
*C* (%, *v*/*v*)	*X*_2_	0	25	50	75	100
*t* (min)	*X*_3_	30	60	90	120	150
*T* (°C)	*X*_4_	20	35	50	65	80

**Table 2:** Experimental findings for the four independent variables under investigation and the dependent variable’s responses (eriocitrin content, total phenolic content (TPC), total flavonoid content (TFC), ferric reducing antioxidant power (FRAP) assay, DPPH radical scavenging activity, hydrogen peroxide (H_2_O_2_) scavenging assay, ascorbic acid content, and total carotenoid content (TCC)).

Design Point	Independent Variables				Responses							
	*X*_1_	*X*_2_	*X*_3_	*X*_4_	Eriocitrin (mg/g)	TPC (mg GAE/g)	TFC (mg RtE/g)	FRAP (μmol AAE/g)	DPPH (μmol AAE/g)	H_2_O_2_ (μmol AAE/g)	Ascorbic Acid (mg/g)	TCC (μg CtE/g)
1	3	1	3	4	6.09	5.90	2.47	20.81	18.97	22.48	2.55	9.47
2	3	2	1	3	6.47	13.13	2.66	40.04	23.89	15.70	2.85	59.21
3	2	3	4	3	6.74	13.56	3.66	41.25	23.44	21.86	2.62	64.90
4	2	4	5	4	6.84	12.48	4.45	34.91	21.16	27.77	2.50	54.39
5	3	5	4	2	5.10	6.61	5.65	22.63	23.52	42.46	3.71	14.99
6	4	1	4	5	5.58	12.20	2.81	25.66	14.31	24.19	1.55	6.25
7	4	2	3	1	6.20	11.80	3.08	38.69	26.87	13.96	3.01	39.40
8	1	3	3	2	6.52	41.52	4.14	113.19	28.70	21.94	3.04	57.83
9	1	4	4	1	6.45	51.24	4.86	128.89	30.31	29.37	3.70	25.55
10	1	5	1	4	5.69	40.57	5.93	120.67	23.08	34.48	3.66	18.83
11	1	1	2	3	6.10	40.15	3.75	113.30	23.59	18.29	3.17	11.63
12	1	2	5	5	6.95	44.50	4.10	85.51	17.82	17.29	1.20	50.39
13	4	3	2	4	6.86	23.92	4.40	62.06	24.27	22.06	3.01	36.59
14	3	4	2	5	6.42	13.41	6.61	32.21	18.51	29.59	3.07	45.29
15	2	5	3	5	6.09	11.77	7.09	38.77	20.33	41.80	3.58	10.47
16	2	1	1	1	6.19	5.13	4.02	21.21	18.27	20.62	3.17	5.38
17	2	2	2	2	6.20	11.18	3.48	37.81	23.26	19.28	2.79	60.25
18	3	3	5	1	6.54	10.31	4.23	29.25	20.89	14.15	3.01	55.99
19	4	4	1	2	6.49	22.15	5.25	40.28	21.79	31.01	3.12	9.91
20	4	5	5	3	5.42	9.60	6.45	36.88	19.84	38.38	3.50	3.34

**Table 3:** Coded values of the four independent variables under investigation and the actual concentration of phenolic compounds, represented in mg/g d*w*.

Design Point	Independent Variables				Responses (mg/g)								
	*X*_1_	*X*_2_	*X*_3_	*X*_4_	NCA	CA	CGA	CFA	SA	LG	KR	KG	HES
1	3	1	3	4	0.17	0.22	0.66	0.07	0.07	0.14	0.27	0.41	2.66
2	3	2	1	3	0.16	0.20	0.76	0.09	0.07	0.16	0.26	0.44	2.91
3	2	3	4	3	0.13	0.09	0.73	0.08	0.06	0.16	0.30	0.44	3.74
4	2	4	5	4	0.15	0.04	0.68	0.07	0.03	0.16	0.29	0.44	4.63
5	3	5	4	2	0.04	0.31	0.40	0.04	0.04	0.13	0.21	0.38	2.75
6	4	1	4	5	0.14	0.19	0.51	0.07	0.05	0.13	0.23	0.39	2.26
7	4	2	3	1	0.15	0.17	0.72	0.09	0.06	0.15	0.28	0.42	2.62
8	1	3	3	2	0.13	0.11	0.72	0.08	0.05	0.16	0.31	0.43	3.64
9	1	4	4	1	0.12	0.16	0.64	0.06	0.03	0.15	0.28	0.42	3.82
10	1	5	1	4	0.06	0.39	0.47	0.06	0.04	0.13	0.22	0.40	3.55
11	1	1	2	3	0.17	0.21	0.72	0.08	0.06	0.14	0.26	0.40	3.04
12	1	2	5	5	0.14	0.18	0.50	0.08	0.05	0.17	0.30	0.43	4.40
13	4	3	2	4	0.12	0.11	0.71	0.08	0.06	0.16	0.31	0.43	4.37
14	3	4	2	5	0.14	0.32	0.65	0.07	0.03	0.16	0.33	0.42	6.44
15	2	5	3	5	0.02	0.37	0.46	0.04	0.01	0.14	0.27	0.41	3.64
16	2	1	1	1	0.18	0.21	0.76	0.09	0.06	0.14	0.26	0.44	3.09
17	2	2	2	2	0.16	0.17	0.74	0.09	0.05	0.15	0.27	0.43	2.58
18	3	3	5	1	0.13	0.09	0.72	0.08	0.05	0.16	0.30	0.42	3.43
19	4	4	1	2	0.11	0.06	0.63	0.07	0.03	0.15	0.30	0.43	3.53
20	4	5	5	3	0.05	0.36	0.43	0.06	0.03	0.13	0.27	0.41	2.89

NCA: Neochlorogenic acid; CA: catechin; CGA: chlorogenic acid; CFA: caffeic acid; SA: syringic acid; LG: luteolin 7-glycoside; KR: kaempferol 3-*O*-β-rutinoside; KG: kaempferol 3-glycoside; HES: hesperidin.

**Table 4:** Mathematical models created using RSM were used to optimize the extraction of Citrus peels from hydroethanolic solutions using various techniques. The models contained only significant terms for dependent variable responses (eriocitrin content, total phenolic content (TPC), total flavonoid content (TFC), ferric reducing antioxidant power (FRAP) assay, DPPH radical scavenging activity, hydrogen peroxide (H_2_O_2_) scavenging assay, ascorbic acid content, and total carotenoid content (TCC)).

Responses	Second-order Polynomial Equations (Models)	R^2^	*p*	Equation
Eriocitrin	*Y* = 5.78 + 0.05*X*_1_ − 0.65*X*_2_ + 1*X*_3_ − 0.08*X*_4_ + 0.08*X*_1_^2^ − 0.01*X*_2_^2^ + 0.1*X*_3_^2^ − 0.13*X*_4_^2^ + 0.01*X*_1_*X*_2_ − 0.31*X*_1_*X*_3_ + 0.13*X*_1_*X*_4_ − 0.11*X*_2_*X*_3_ + 0.25*X*_2_*X*_4_ − 0.09*X*_3_*X*_4_	0.9934	0.0002	(8)
TPC	*Y* = 60 *−* 51.54*X*_1_ + 5.29*X*_2_ + 3.07*X*_3_ + 6.89*X*_4_ + 9.64*X*_1_^2^ − 0.58*X*_2_^2^ + 0.65*X*_3_^2^ + 0.69*X*_4_^2^ + 0.09*X*_1_*X*_2_ − 1.97*X*_1_*X*_3_ − 0.03*X*_1_*X*_4_ + 0.73*X*_2_*X*_3_ − 1.3*X*_2_*X*_4_ − 1.99*X*_3_*X*_4_	0.9564	0.0164	(9)
TFC	*Y* = 8.82 − 1.84*X*_1_ − 1.23*X*_2_ + 0.5*X*_3_ − 2.02*X*_4_ + 0.05*X*_1_^2^ + 0.16*X*_2_^2^ + 0.002*X*_3_^2^ + 0.21*X*_4_^2^ + 0.38*X*_1_*X*_2_ − 0.007*X*_1_*X*_3_ + 0.17*X*_1_*X*_4_ − 0.14*X*_2_*X*_3_ + 0.17*X*_2_*X*_4_ − 0.03*X*_3_*X*_4_	0.9523	0.0202	(10)
FRAP	*Y* = 160.55 − 132.65*X*_1_ + 5.35*X*_2_ + 6.77*X*_3_ + 29.87*X*_4_ + 24.68*X*_1_^2^ − 1.08*X*_2_^2^ + 0.57*X*_3_^2^ − 0.8*X*_4_^2^ − 2.01*X*_1_*X*_2_ − 2.45*X*_1_*X*_3_ − 0.25*X*_1_*X*_4_ + 3.42*X*_2_*X*_3_ − 1.21*X*_2_*X*_4_ − 6.22*X*_3_*X*_4_	0.9623	0.0117	(11)
DPPH	*Y* = 9.9 − 3.04*X*_1_ + 5.88*X*_2_ + 2.23*X*_3_ + 5.75*X*_4_ + 1.56*X*_1_^2^ − 0.88*X*_2_^2^ − 0.83*X*_3_^2^ − 0.36*X*_4_^2^ − 0.99*X*_1_*X*_2_ − 0.28*X*_1_*X*_3_ − 0.74*X*_1_*X*_4_ + 1.28*X*_2_*X*_3_ − 0.4*X*_2_*X*_4_ − 0.44*X*_3_*X*_4_	0.9614	0.0124	(12)
H_2_O_2_	*Y* = 26.38 + 1.6*X*_1_ − 10.44*X*_2_ + 5.48*X*_3_ − 4.83*X*_4_ − 0.85*X*_1_^2^ + 2.85*X*_2_^2^ − 0.76*X*_3_^2^ − 0.17*X*_4_^2^ + 0.49*X*_1_*X*_2_ − 0.66*X*_1_*X*_3_ + 1.21*X*_1_*X*_4_ − 0.64*X*_2_*X*_3_ − 0.08*X*_2_*X*_4_ + 0.92*X*_3_*X*_4_	0.9563	0.0165	(13)
Ascorbic acid	*Y* = 3.96 − 0.26*X*_1_ − 1.51*X*_2_ + 0.8*X*_3_ + 0.12*X*_4_ + 0.08*X*_1_^2^ + 0.2*X*_2_^2^ − 0.02*X*_3_^2^ − 0.07*X*_4_^2^ + 0.01*X*_1_*X*_2_ − 0.13*X*_1_*X*_3_ + 0.06*X*_1_*X*_4_ + 0.03*X*_2_*X*_3_ + 0.13*X*_2_*X*_4_ − 0.16*X*_3_*X*_4_	0.9725	0.0056	(14)
TCC	*Y* = −108.57 + 25.91*X*_1_ + 135.53*X*_2_ − 50.15*X*_3_ + 20.91*X*_4_ − 7*X*_1_^2^ − 20.5*X*_2_^2^ − 2.16*X*_3_^2^ + 3.14*X*_4_^2^ − 2.89*X*_1_*X*_2_ + 11.09*X*_1_*X*_3_ − 7.5*X*_1_*X*_4_ + 6.6*X*_2_*X*_3_ − 7.44*X*_2_*X*_4_ + 2.76*X*_3_*X*_4_	0.9805	0.0024	(15)

**Table 5:** Maximum predicted responses (eriocitrin content, total phenolic content (TPC), total flavonoid content (TFC), ferric reducing antioxidant power (FRAP) assay, DPPH radical scavenging activity, hydrogen peroxide (H_2_O_2_) scavenging assay, ascorbic acid content, and total carotenoid content (TCC)) and optimum extraction conditions for the dependent variables using different extraction methods and hydroethanolic solutions.

Responses	Optimal Conditions				
	Maximum Predicted Response	Technique (*X*_1_)	*C* (%, *v*/*v*) (*X*_2_)	*t* (min) (*X*_3_)	*T* (°C) (*X*_4_)
Eriocitrin (mg/g)	7.2 ± 0.2	ST (1)	50 (3)	120 (4)	50 (3)
TPC (mg GAE/g)	51 ± 14	ST (1)	75 (4)	120 (4)	20 (1)
TFC (mg RtE/g)	7 ± 1	PEF + ST (2)	100 (5)	60 (2)	80 (5)
FRAP (μmol AAE/g)	128 ± 33	ST (1)	75 (4)	120 (4)	20 (1)
DPPH (μmol AAE/g)	30 ± 3	ST (1)	75 (4)	120 (4)	35 (2)
H_2_O_2_ (μmol AAE/g)	45 ± 9	US + ST (3)	100 (5)	120 (4)	65 (4)
Ascorbic acid (mg/g)	3.9 ± 0.5	PEF + ST (2)	100 (5)	120 (4)	35 (2)
TCC (μg CtE/g)	81 ± 12	PEF + ST (2)	50 (3)	120 (4)	65 (4)

**Table 6:** Maximum desirability for all variables (eriocitrin content, total phenolic content (TPC), total flavonoid content (TFC), ferric reducing antioxidant power (FRAP) assay, DPPH radical scavenging activity, hydrogen peroxide (H_2_O_2_) scavenging assay, ascorbic acid content and total carotenoid content (TCC)) using the partial least squares (PLS) prediction profiler under the optimal extraction conditions (*X*_1_:1, *X*_2_:4, *X*_3_:5, and *X*_4_:1).

Variables	PLS Model Values	Experimental Values
Eriocitrin (mg/g)	6.72	6.6 ± 0.4
TPC (mg GAE/g)	47.75	45 ± 1
TFC (mg RtE/g)	5.43	5.3 ± 0.5
FRAP (μmol AAE/g)	119.54	112 ± 4
DPPH (μmol AAE/g)	28.97	28.0 ± 0.8
H_2_O_2_ (μmol AAE/g)	26.81	26.0 ± 0.7
Ascorbic acid (mg/g)	3.49	3.4 ± 0.2
TCC (μg CtE/g)	43.45	41 ± 1

**Table 7:** Phenolic compounds under optimal extraction conditions.

Phenolic Compounds	Optimal Extract (mg/g)
Neochlorogenic acid	0.17 ± 0.01
Catechin	0.35 ± 0.02
Chlorogenic acid	0.64 ± 0.03
Caffeic acid	0.07 ± 0.01
Syringic acid	0.06 ± 0.01
Luteolin 7-glycoside	0.16 ± 0.01
Kaempferol 3-*O*-β-rutinoside	0.32 ± 0.02
Kaempferol 3-glycoside	0.44 ± 0.03
Hesperidin	6.3 ± 0.4

## Data Availability

All related data and methods are presented in this paper. Additional inquiries should be addressed to the corresponding author.

## Supplementary Materials

The following supporting information can be downloaded at: https://www.mdpi.com/article/10.3390/antiox12081605/s1.
