# Supplementary material for: Response Surface Optimization for the Enhancement of the Extraction of Bioactive Compounds from Citrus limon Peel
Source: Antioxidants (Basel). 2023 Aug 12;12(8):1605. doi: 10.3390/antiox12081605 (PMC10451340; doi:10.3390/antiox12081605)
Supplement: Supplementary file 1 [file antioxidants-12-01605-s001.zip › antioxidants-2543255-supplementary.pdf]

Supplementary material

Response surface optimization for the enhancement of the extraction of bioactive compounds from *Citrus limon* peel

Theodoros Chatzimitakos, Vassilis Athanasiadis *, Konstantina Kotsou, Eleni Bozinou and Stavros I. Lalas

Department of Food Science & Nutrition, University of Thessaly, Terma N. Temponera str., 43100 Karditsa, Greece; tchatzimitakos@uth.gr (T.C.); kkotsou@agr.uth.gr (K.K.); empozinou@uth.gr (E.B.); slalas@uth.gr (S.I.L.)

* Correspondence: vaathanasiadis@uth.gr; Tel.: +30-24410-64783

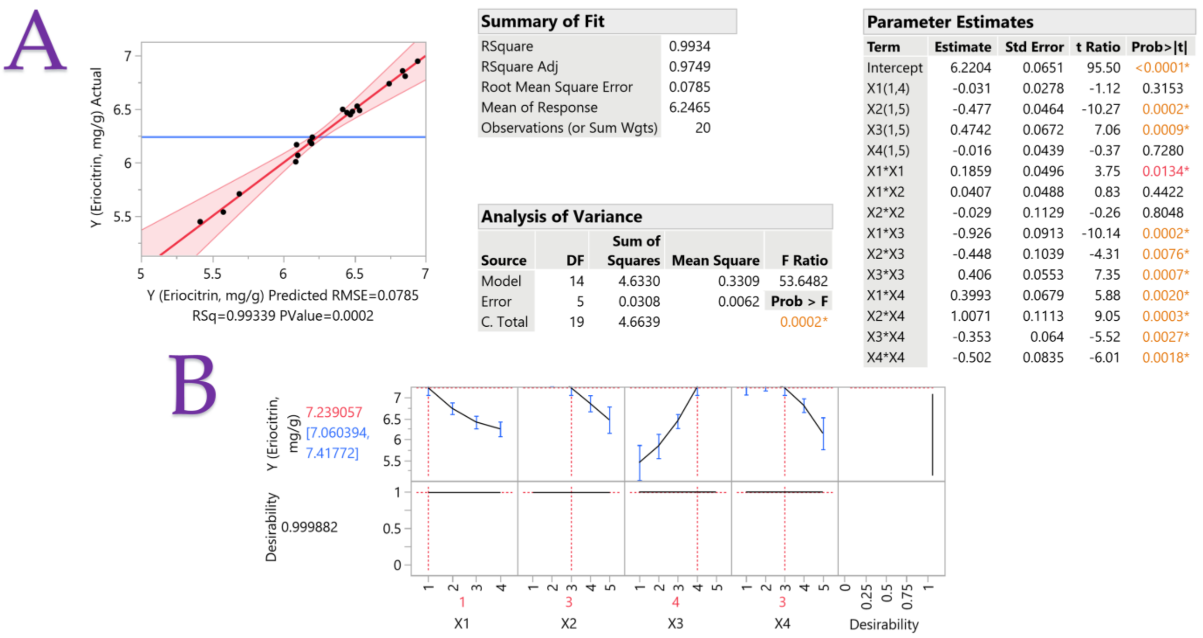

Figure S1. Plots A and B display the actual response versus the predicted response (Eriocitrin, mg/g) for the optimization of Citrus peel by-product extracts carried out with hydroethanolic solutions and different extraction methods, as well as the desirability function. Asterisks and colored values denote statistically significant values, while inset tables include statistics relevant to the evaluation of the resulting model.

A

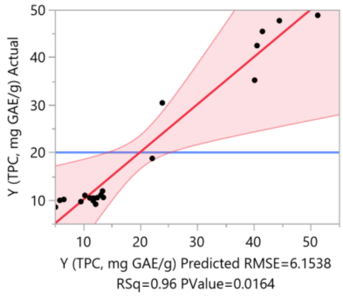

Summary of Fit

RSquare	0.9564
RSquare Adj	0.8343
Root Mean Square Error	6.1538
Mean of Response	20.057
Observations (or Sum Wgts)	20

Analysis of Variance

Source	DF	Sum of Squares	Mean Square	F Ratio
Model	14	4152.2	296.584	7.8317
Error	5	189.35	37.870	**Prob > F**
C. Total	19	4341.5		0.0164*

Parameter Estimates

Term	Estimate	Std Error	t Ratio	Prob>|t|
Intercept	6.7149	5.1034	1.32	0.2453
X1(1,4)	-13.58	2.1802	-6.23	0.0016*
X2(1,5)	0.6731	3.6375	0.19	0.8605
X3(1,5)	-3.461	5.264	-0.66	0.5399
X4(1,5)	2.2336	3.4388	0.65	0.5447
X1*X1	21.687	3.8892	5.58	0.0026*
X1*X2	0.2675	3.821	0.07	0.9469
X2*X2	-2.317	8.844	-0.26	0.8038
X1*X3	-5.901	7.1548	-0.82	0.4470
X2*X3	2.9116	8.1413	0.36	0.7352
X3*X3	2.6066	4.3296	0.60	0.5734
X1*X4	-0.079	5.3216	-0.01	0.9887
X2*X4	-5.18	8.7233	-0.59	0.5785
X3*X4	-7.971	5.0107	-1.59	0.1725
X4*X4	2.7732	6.5453	0.42	0.6894

B

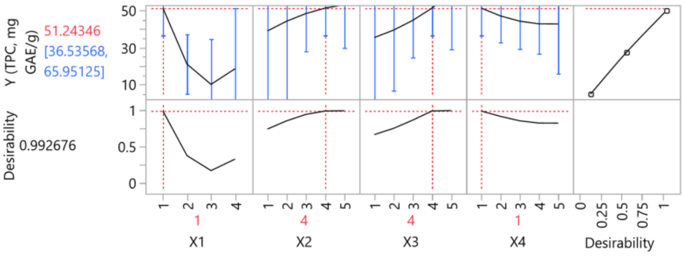

Figure S2. Plots A and B display the actual response versus the predicted response (Total phenolic content – TPC, mg GAE/g) for the optimization of Citrus peel by-product extracts carried out with hydroethanolic solutions and different extraction methods, as well as the desirability function. Asterisks and colored values denote statistically significant values, while inset tables include statistics relevant to the evaluation of the resulting model.

A

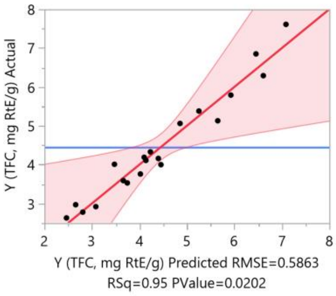

Summary of Fit	
RSquare	0.9523
RSquare Adj	0.8186
Root Mean Square Error	0.5863
Mean of Response	4.4545
Observations (or Sum Wgts)	20

Analysis of Variance

Source	DF	Sum of Squares	Mean Square	F Ratio
Model	14	34.2797	2.4486	7.1238
Error	5	1.7186	0.3437	Prob > F
C. Total	19	35.9983		0.0202*

Parameter Estimates

Term	Estimate	Std Error	t Ratio	Prob>|t|
Intercept	3.6692	0.4862	7.55	0.0006*
X1(1,4)	-0.008	0.2077	-0.04	0.9716
X2(1,5)	1.5437	0.3465	4.45	0.0067*
X3(1,5)	-0.05	0.5015	-0.10	0.9242
X4(1,5)	0.2098	0.3276	0.64	0.5502
X1*X1	0.1035	0.3705	0.28	0.7912
X1*X2	1.1294	0.364	3.10	0.0268*
X2*X2	0.6489	0.8426	0.77	0.4760
X1*X3	-0.02	0.6816	-0.03	0.9782
X2*X3	-0.57	0.7756	-0.73	0.4956
X3*X3	0.0072	0.4125	0.02	0.9868
X1*X4	0.4966	0.507	0.98	0.3723
X2*X4	0.6859	0.8311	0.83	0.4467
X3*X4	-0.123	0.4774	-0.26	0.8076
X4*X4	0.857	0.6236	1.37	0.2277

B

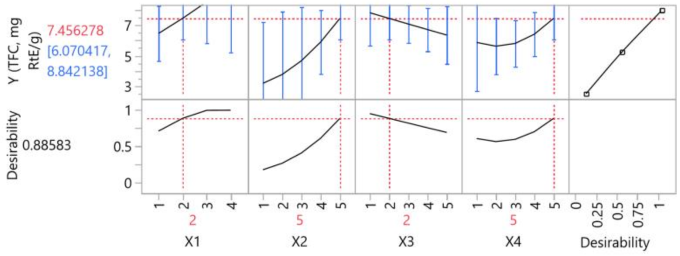

Figure S3. Plots A and B display the actual response versus the predicted response (Total flavonoid content – TFC, mg RtE/g) for the optimization of Citrus peel by-product extracts carried out with hydroethanolic solutions and different extraction methods, as well as the desirability function. Asterisks and colored values denote statistically significant values, while inset tables include statistics relevant to the evaluation of the resulting model.

A

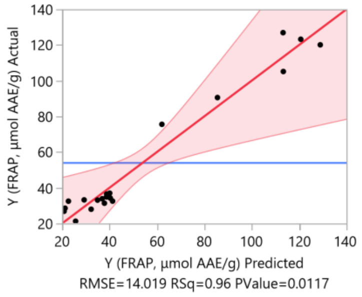

Summary of Fit	
RSquare	0.9623
RSquare Adj	0.8569
Root Mean Square Error	14.019
Mean of Response	54.202
Observations (or Sum Wgts)	20

Analysis of Variance

Source	DF	Sum of Squares	Mean Square	F Ratio
Model	14	25,113	1793.8	9.1267
Error	5	982.71	196.54	Prob > F
C. Total	19	26,096		0.0117*

Parameter Estimates

Term	Estimate	Std Error	t Ratio	Prob>|t|
Intercept	25.893	11.626	2.23	0.0764
X1(1,4)	-35.08	4.9669	-7.06	0.0009*
X2(1,5)	0.877	8.2867	0.11	0.9198
X3(1,5)	-8.605	11.992	-0.72	0.5052
X4(1,5)	4.2694	7.834	0.54	0.6092
X1*X1	55.531	8.8603	6.27	0.0015*
X1*X2	-6.045	8.7048	-0.69	0.5184
X2*X2	-4.332	20.148	-0.22	0.8382
X1*X3	-7.345	16.3	-0.45	0.6711
X2*X3	13.695	18.547	0.74	0.4935
X3*X3	2.2882	9.8634	0.23	0.8257
X1*X4	-0.752	12.123	-0.06	0.9530
X2*X4	-4.857	19.873	-0.24	0.8166
X3*X4	-24.87	11.415	-2.18	0.0812
X4*X4	-3.207	14.911	-0.22	0.8382

B

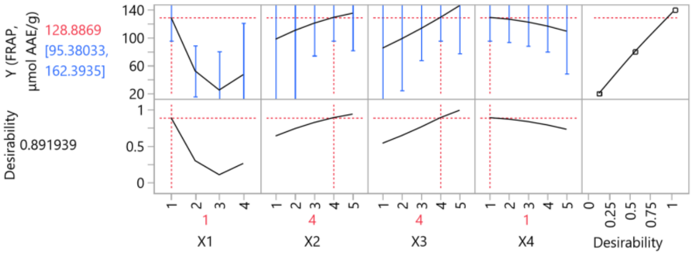

Figure S4. Plots A and B display the actual response versus the predicted response (FRAP, µmol AAE/g) for the optimization of Citrus peel by-product extracts carried out with hydroethanolic solutions and different extraction methods, as well as the desirability function. Asterisks and colored values denote statistically significant values, while inset tables include statistics relevant to the evaluation of the resulting model.

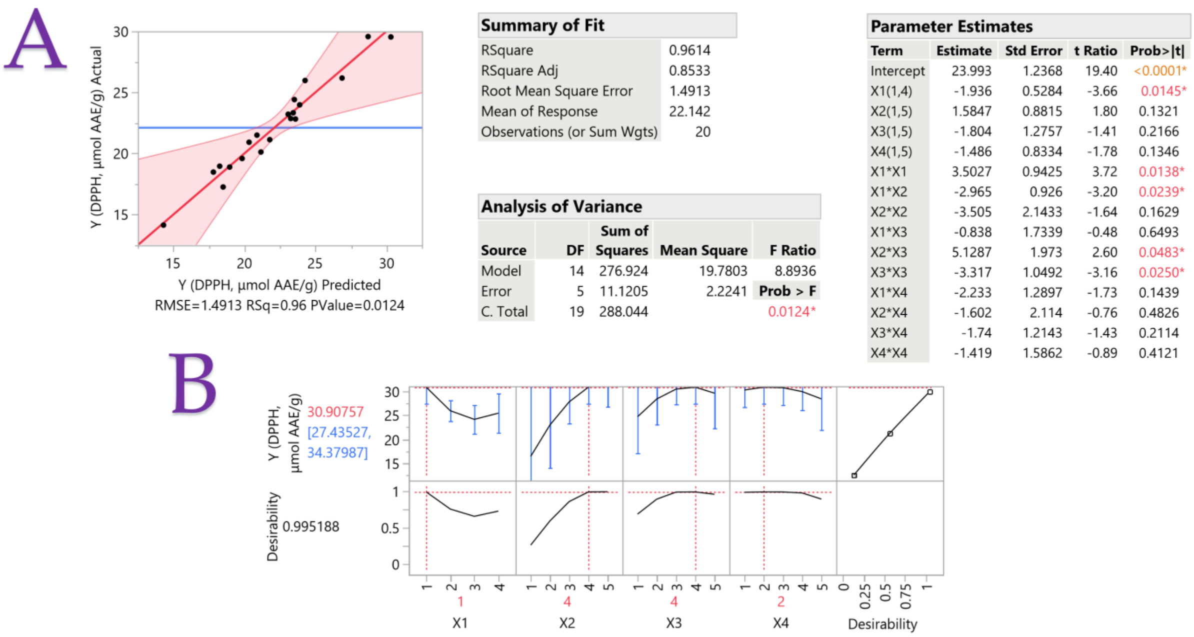

Summary of Fit

RSquare	0.9614
RSquare Adj	0.8533
Root Mean Square Error	1.4913
Mean of Response	22.142
Observations (or Sum Wgts)	20

Analysis of Variance

Source	DF	Sum of Squares	Mean Square	F Ratio
Model	14	276.924	19.7803	8.8936
Error	5	11.1205	2.2241	Prob > F
C. Total	19	288.044		0.0124*

Parameter Estimates

Term	Estimate	Std Error	t Ratio	Prob>|t|
Intercept	23.993	1.2368	19.40	<0.0001*
X1(1,4)	-1.936	0.5284	-3.66	0.0145*
X2(1,5)	1.5847	0.8815	1.80	0.1321
X3(1,5)	-1.804	1.2757	-1.41	0.2166
X4(1,5)	-1.486	0.8334	-1.78	0.1346
X1*X1	3.5027	0.9425	3.72	0.0138*
X1*X2	-2.965	0.926	-3.20	0.0239*
X2*X2	-3.505	2.1433	-1.64	0.1629
X1*X3	-0.838	1.7339	-0.48	0.6493
X2*X3	5.1287	1.973	2.60	0.0483*
X3*X3	-3.317	1.0492	-3.16	0.0250*
X1*X4	-2.233	1.2897	-1.73	0.1439
X2*X4	-1.602	2.114	-0.76	0.4826
X3*X4	-1.74	1.2143	-1.43	0.2114
X4*X4	-1.419	1.5862	-0.89	0.4121

Figure S5. Plots A and B display the actual response versus the predicted response (DPPH, μmol AAE/g) for the optimization of Citrus peel by-product extracts carried out with hydroethanolic solutions and different extraction methods, as well as the desirability function. Asterisks and colored values denote statistically significant values, while inset tables include statistics relevant to the evaluation of the resulting model.

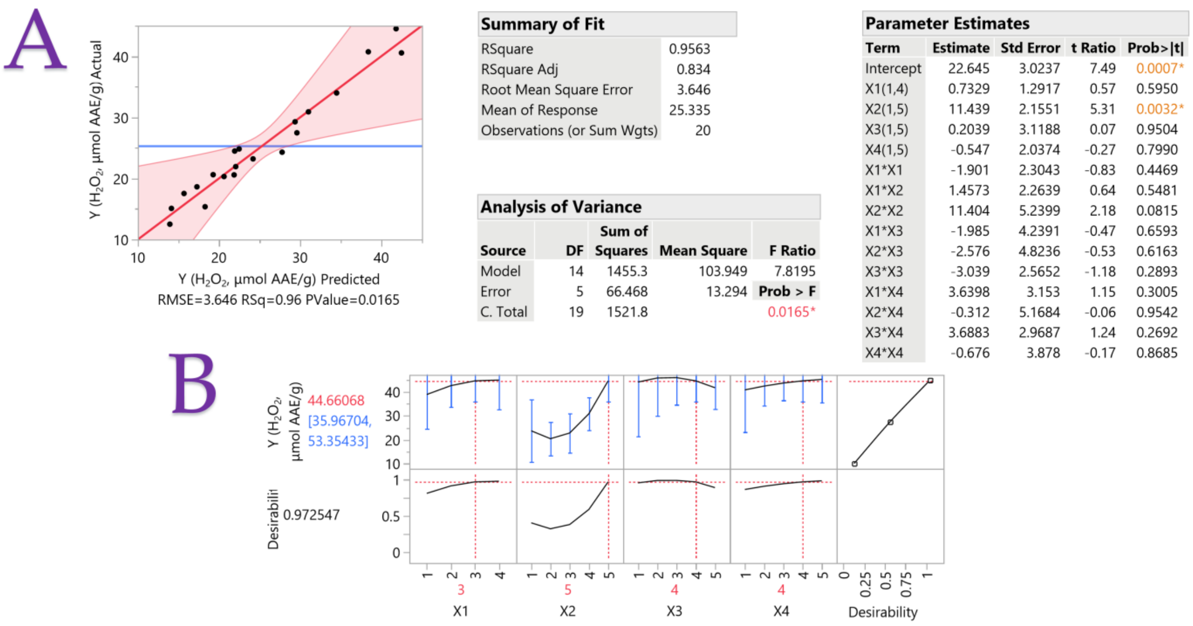

Summary of Fit

RSquare	0.9563
RSquare Adj	0.834
Root Mean Square Error	3.646
Mean of Response	25.335
Observations (or Sum Wgts)	20

Analysis of Variance

Source	DF	Sum of Squares	Mean Square	F Ratio
Model	14	1455.3	103.949	7.8195
Error	5	66.468	13.294	Prob > F
C. Total	19	1521.8		0.0165*

Parameter Estimates

Term	Estimate	Std Error	t Ratio	Prob>|t|
Intercept	22.645	3.0237	7.49	0.0007*
X1(1,4)	0.7329	1.2917	0.57	0.5950
X2(1,5)	11.439	2.1551	5.31	0.0032*
X3(1,5)	0.2039	3.1188	0.07	0.9504
X4(1,5)	-0.547	2.0374	-0.27	0.7990
X1*X1	-1.901	2.3043	-0.83	0.4469
X1*X2	1.4573	2.2639	0.64	0.5481
X2*X2	11.404	5.2399	2.18	0.0815
X1*X3	-1.985	4.2391	-0.47	0.6593
X2*X3	-2.576	4.8236	-0.53	0.6163
X3*X3	-3.039	2.5652	-1.18	0.2893
X1*X4	3.6398	3.153	1.15	0.3005
X2*X4	-0.312	5.1684	-0.06	0.9542
X3*X4	3.6883	2.9687	1.24	0.2692
X4*X4	-0.676	3.878	-0.17	0.8685

Figure S6. Plots A and B display the actual response versus the predicted response (H_2O_2, μmol AAE/g) for the optimization of Citrus peel by-product extracts carried out with hydroethanolic solutions and different extraction methods, as well as the desirability function. Asterisks and colored values denote statistically significant values, while inset tables include statistics relevant to the evaluation of the resulting model.

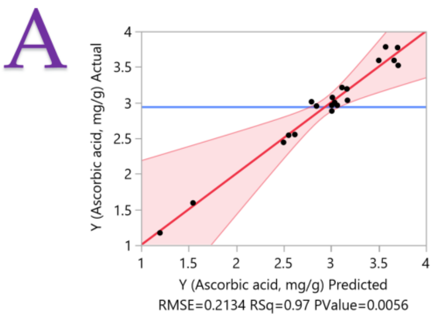

Summary of Fit	
RSquare	0.9725
RSquare Adj	0.8953
Root Mean Square Error	0.2134
Mean of Response	2.94
Observations (or Sum Wgts)	20

Analysis of Variance

Source	DF	Sum of Squares	Mean Square	F Ratio
Model	14	8.0391	0.5742	12.6092
Error	5	0.2277	0.0455	Prob > F
C. Total	19	8.2668		0.0056*

Parameter Estimates

Term	Estimate	Std Error	t Ratio	Prob>|t|
Intercept	2.6117	0.177	14.76	<0.0001*
X1(1,4)	-0.006	0.0756	-0.09	0.9353
X2(1,5)	0.3598	0.1261	2.85	0.0357*
X3(1,5)	-0.112	0.1825	-0.61	0.5666
X4(1,5)	-0.402	0.1192	-3.37	0.0200*
X1*X1	0.1884	0.1349	1.40	0.2213
X1*X2	0.0309	0.1325	0.23	0.8250
X2*X2	0.7905	0.3067	2.58	0.0496*
X1*X3	-0.383	0.2481	-1.54	0.1831
X2*X3	0.1178	0.2823	0.42	0.6939
X3*X3	-0.095	0.1501	-0.63	0.5559
X1*X4	0.1924	0.1845	1.04	0.3449
X2*X4	0.5264	0.3025	1.74	0.1423
X3*X4	-0.641	0.1738	-3.69	0.0141*
X4*X4	-0.261	0.227	-1.15	0.3030

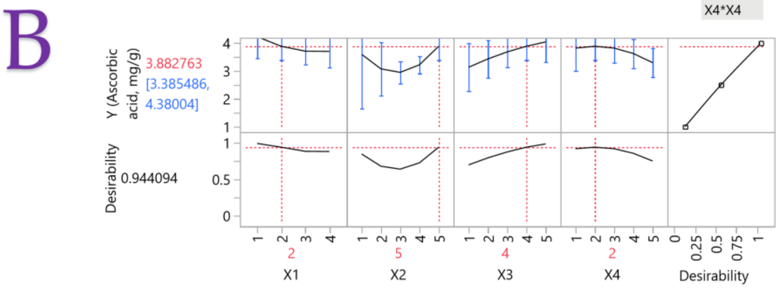

Figure S7. Plots A and B display the actual response versus the predicted response (Ascorbic acid, mg/g) for the optimization of Citrus peel by-product extracts carried out with hydroethanolic solutions and different extraction methods, as well as the desirability function. Asterisks and colored values denote statistically significant values, while inset tables include statistics relevant to the evaluation of the resulting model.

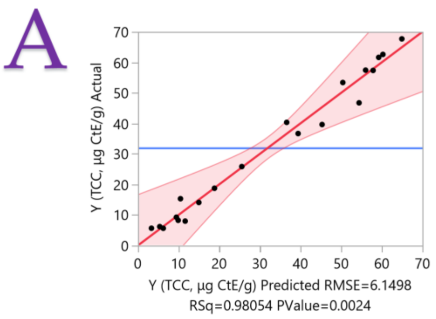

Summary of Fit	
RSquare	0.9805
RSquare Adj	0.926
Root Mean Square Error	6.1498
Mean of Response	32.004
Observations (or Sum Wgts)	20

Analysis of Variance

Source	DF	Sum of Squares	Mean Square	F Ratio
Model	14	9527.7	680.551	17.9944
Error	5	189.10	37.820	Prob > F
C. Total	19	9716.8		0.0024*

Parameter Estimates

Term	Estimate	Std Error	t Ratio	Prob>|t|
Intercept	78.18	5.1001	15.33	<0.0001*
X1(1,4)	-10.43	2.1788	-4.79	0.0049*
X2(1,5)	5.5565	3.6351	1.53	0.1869
X3(1,5)	-14.61	5.2606	-2.78	0.0390*
X4(1,5)	13.85	3.4365	4.03	0.0100*
X1*X1	-15.73	3.8867	-4.05	0.0099*
X1*X2	-8.654	3.8185	-2.27	0.0728
X2*X2	-82.01	8.8382	-9.28	0.0002*
X1*X3	33.261	7.1501	4.65	0.0056*
X2*X3	26.404	8.136	3.25	0.0228*
X3*X3	-8.63	4.3267	-1.99	0.1027
X1*X4	-22.51	5.3181	-4.23	0.0082*
X2*X4	-29.77	8.7176	-3.41	0.0189*
X3*X4	11.022	5.0074	2.20	0.0790
X4*X4	12.554	6.5411	1.92	0.1130

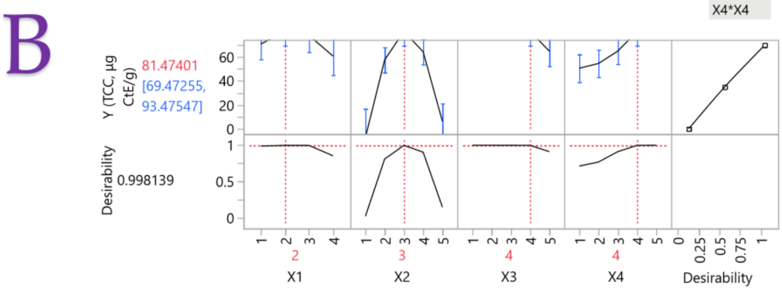

Figure S8. Plots A and B display the actual response versus the predicted response (Total carotenoid content – TCC, µg CtE/g) for the optimization of Citrus peel by-product extracts carried out with hydroethanolic solutions and different extraction methods, as well as the desirability function. Asterisks and colored values denote statistically significant values, while inset tables include statistics relevant to the evaluation of the resulting model.

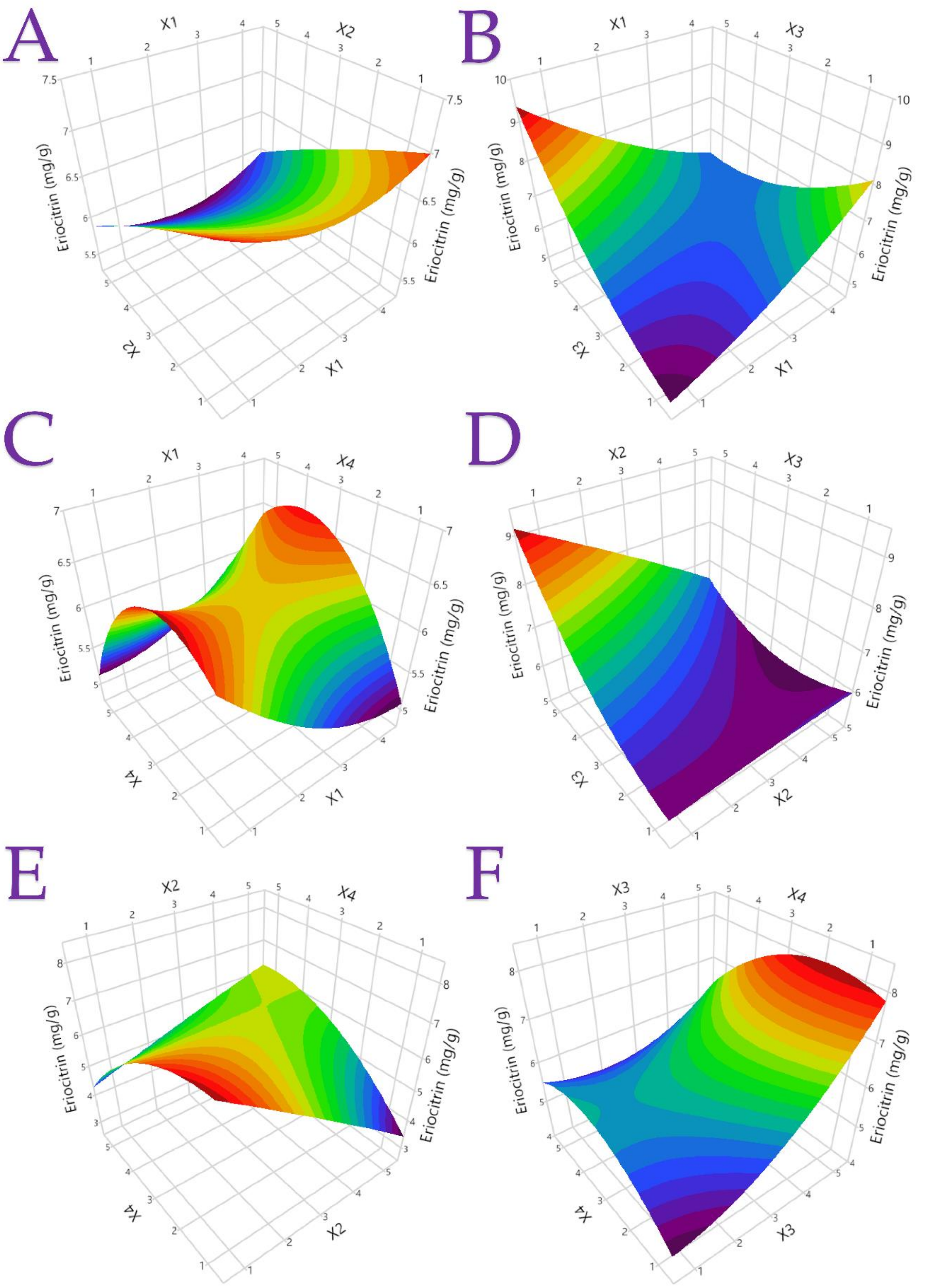

Figure S9. The optimal extraction of Citrus peel by-product extracts using different extraction methods and hydroethanolic solutions is shown in 3D graphs that show the impact of the process variables considered in the response (Eriocitrin, mg/g). Plot (A), covariation of X_1 and X_2; plot (B), covariation of X_1 and X_3; plot (C), covariation of X_1 and X_4; plot (D), covariation of X_2 and X_3; plot (E), covariation of X_2 and X_4; plot (F), covariation of X_3 and X_4.

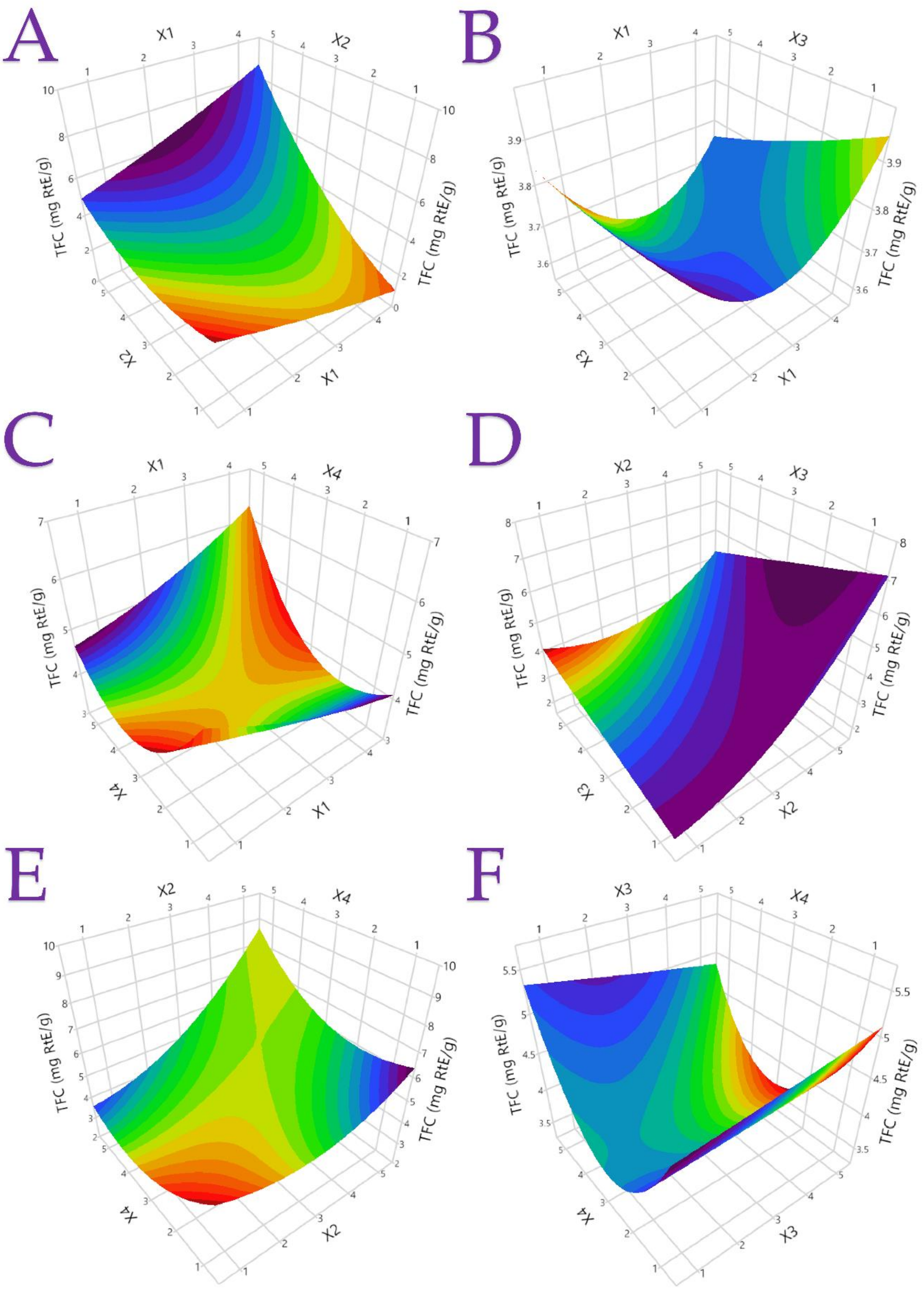

Figure S10. The optimal extraction of Citrus peel by-product extracts using different extraction methods and hydroethanolic solutions is shown in 3D graphs that show the impact of the process variables considered in the response (Total flavonoid content – TFC, mg RtE/g). Plot (A), covariation of X_1 and X_2; plot (B), covariation of X_1 and X_3; plot (C), covariation of X_1 and X_4; plot (D), covariation of X_2 and X_3; plot (E), covariation of X_2 and X_4; plot (F), covariation of X_3 and X_4.

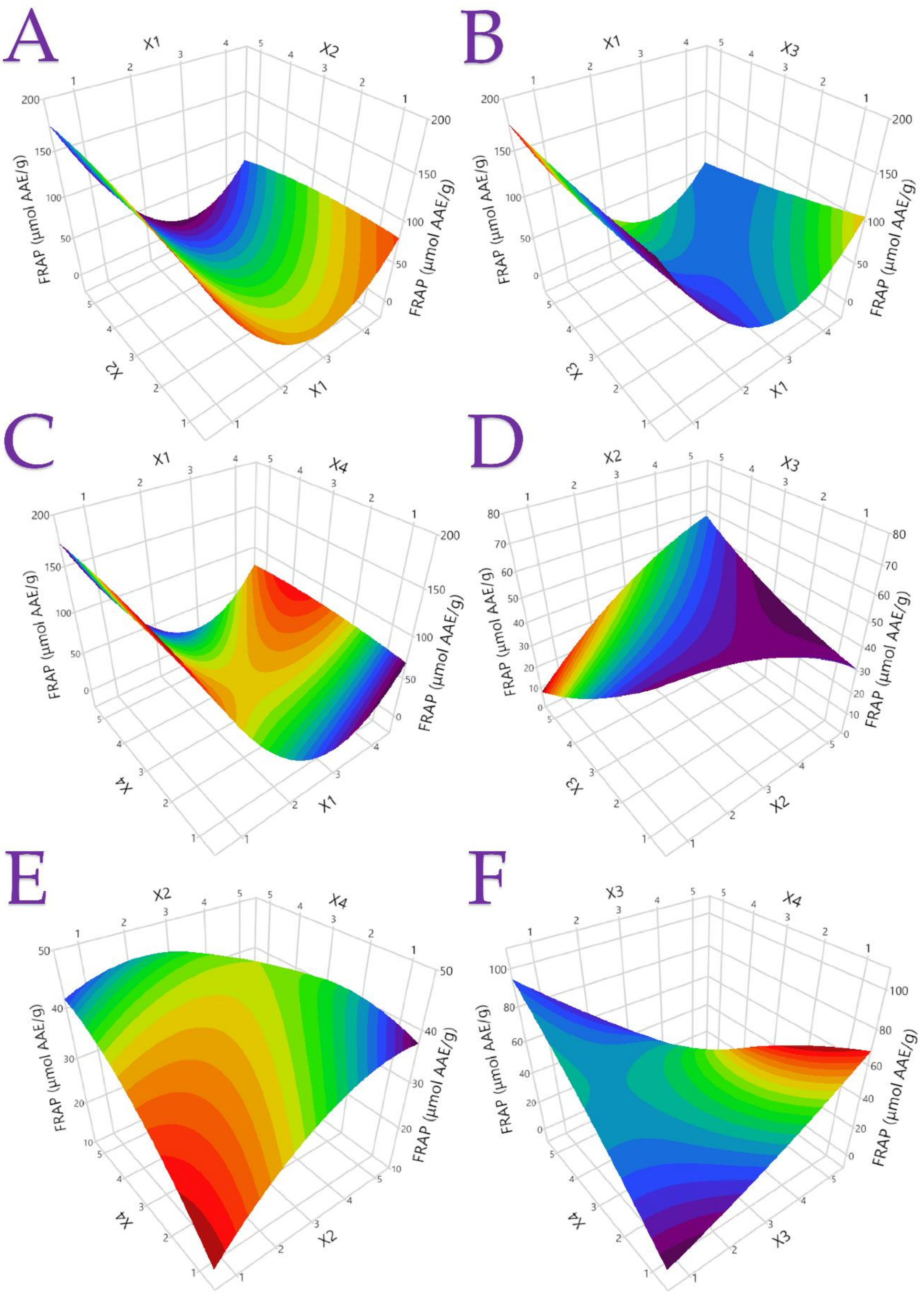

Figure S11. The optimal extraction of Citrus peel by-product extracts using different extraction methods and hydroethanolic solutions is shown in 3D graphs that show the impact of the process variables considered in the response (FRAP, µmol AAE/g). Plot (A), covariation of X_1 and X_2; plot (B), covariation of X_1 and X_3; plot (C), covariation of X_1 and X_4; plot (D), covariation of X_2 and X_3; plot (E), covariation of X_2 and X_4; plot (F), covariation of X_3 and X_4.

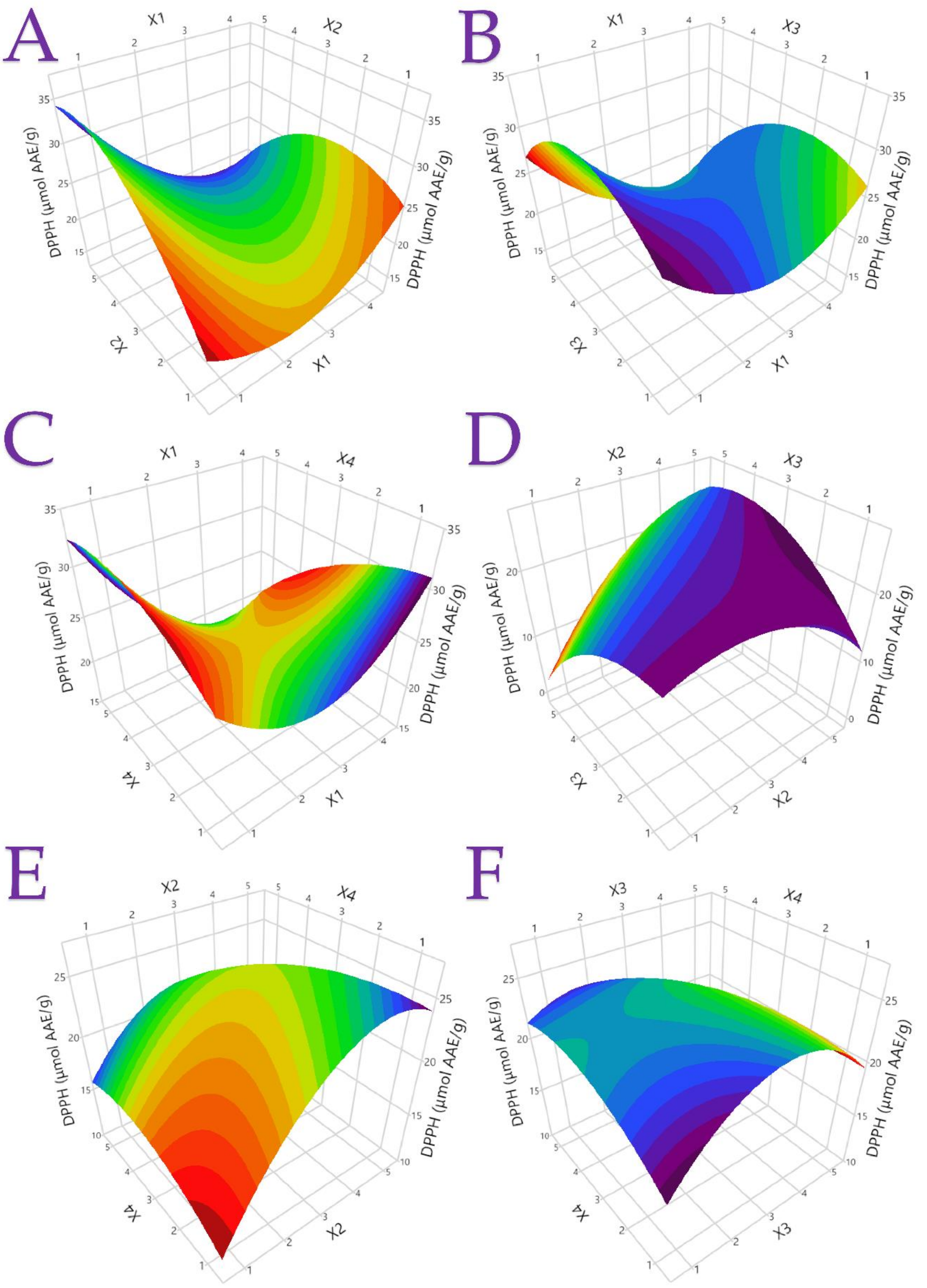

Figure S12. The optimal extraction of Citrus peel by-product extracts using different extraction methods and hydroethanolic solutions is shown in 3D graphs that show the impact of the process variables considered in the response (DPPH, μmol AAE/g). Plot (A), covariation of X_1 and X_2; plot (B), covariation of X_1 and X_3; plot (C), covariation of X_1 and X_4; plot (D), covariation of X_2 and X_3; plot (E), covariation of X_2 and X_4; plot (F), covariation of X_3 and X_4.

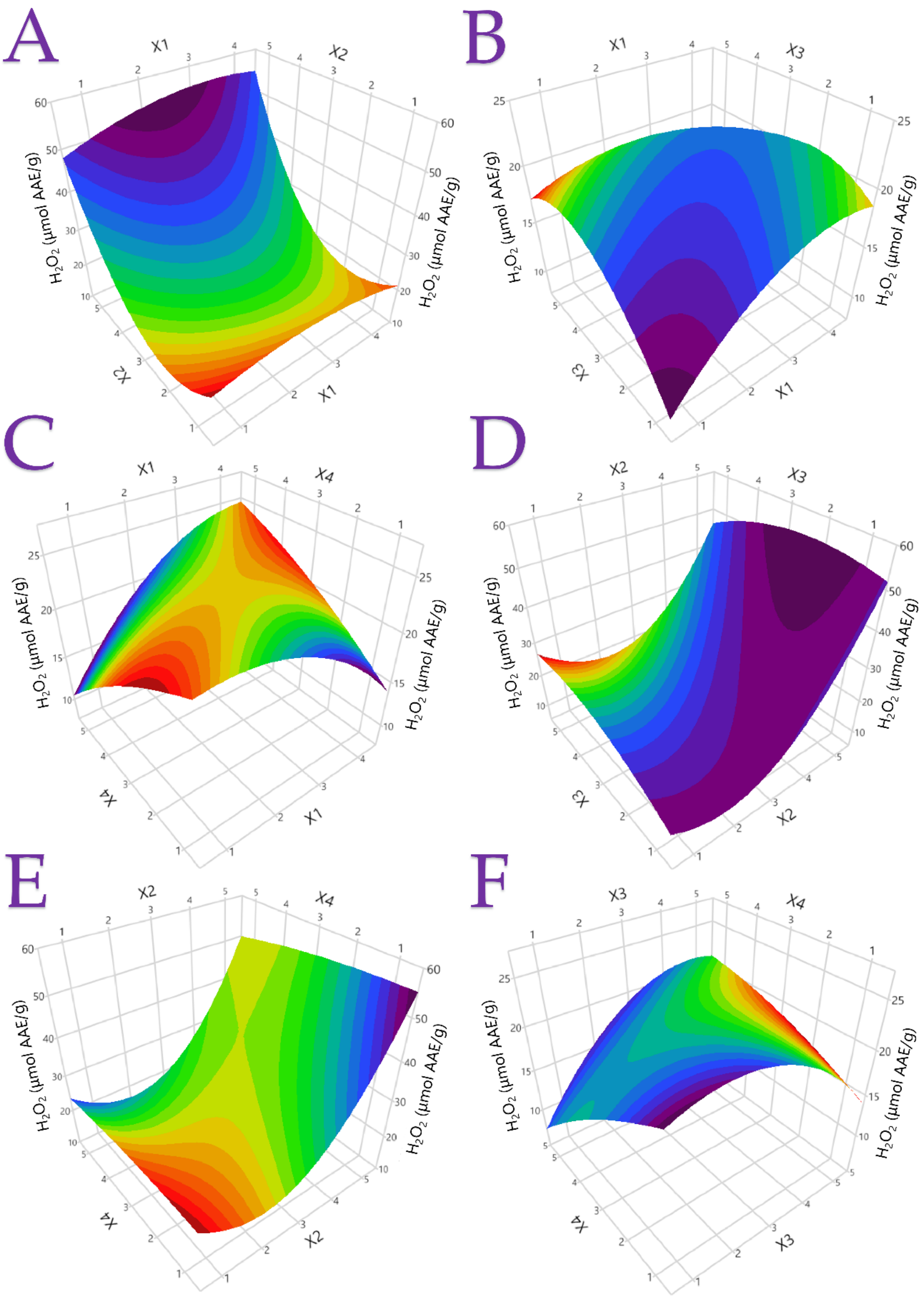

Figure S13. The optimal extraction of Citrus peel by-product extracts using different extraction methods and hydroethanolic solutions is shown in 3D graphs that show the impact of the process variables considered in the response (H_2O_2, μmol AAE/g). Plot (A), covariation of X_1 and X_2; plot (B), covariation of X_1 and X_3; plot (C), covariation of X_1 and X_4; plot (D), covariation of X_2 and X_3; plot (E), covariation of X_2 and X_4; plot (F), covariation of X_3 and X_4.

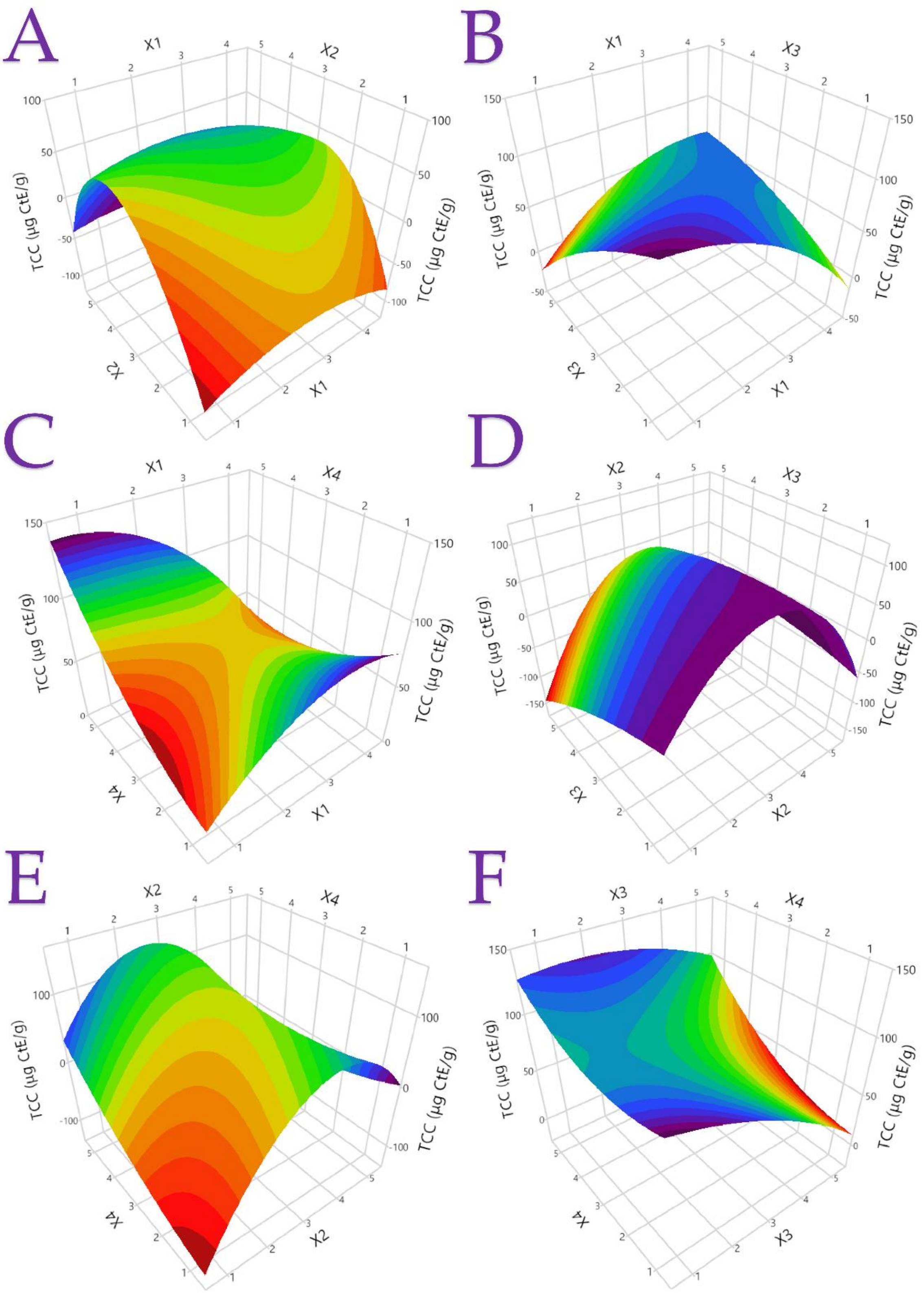

Figure S14. The optimal extraction of Citrus peel by-product extracts using different extraction methods and hydroethanolic solutions is shown in 3D graphs that show the impact of the process variables considered in the response (Total carotenoid content – TCC, µg CtE/g). Plot (A), covariation of X_1 and X_2; plot (B), covariation of X_1 and X_3; plot (C), covariation of X_1 and X_4; plot (D), covariation of X_2 and X_3; plot (E), covariation of X_2 and X_4; plot (F), covariation of X_3 and X_4.
